# Vitamin D supplementation and exercise for improving physical function, body composition and metabolic health in overweight or obese older adults with vitamin D deficiency: a pilot randomized, double-blind, placebo-controlled trial

**DOI:** 10.1007/s00394-022-03038-z

**Published:** 2022-11-04

**Authors:** Jakub Mesinovic, Alexander J. Rodriguez, Mavil May Cervo, Anoohya Gandham, Cecilia L.H. Xu, Costas Glavas, Barbora de Courten, Ayse Zengin, Peter R. Ebeling, David Scott

**Affiliations:** 1 Department of Medicine, School of Clinical Sciences at Monash Health, Faculty of Medicine, Nursing and Health Sciences, Monash University, 246 Clayton Road, Clayton, VIC 3068 Australia; 2 School of Exercise and Nutrition Sciences, Institute for Physical Activity and Nutrition (IPAN), Deakin University, Geelong, Australia; 3 School of Medical and Health Sciences, Edith Cowan University, Perth, WA Australia

**Keywords:** Vitamin D, Exercise, Physical function, Body composition, Metabolic health

## Abstract

**Purpose:**

Vitamin D supplementation may have non-skeletal health benefits and enhance exercise responsiveness, particularly in those with low vitamin D levels. We determined whether, compared with placebo, vitamin D supplementation taken prior to and during a 12-week exercise program improves physical function, body composition or metabolic health, in overweight and obese older adults with vitamin D deficiency.

**Methods:**

Fifty overweight or obese older adults (mean ± SD age: 60 ± 6 years; BMI 30.6 ± 5.7 kg/m^2^) with vitamin D deficiency (25-hydroxyvitamin D [25(OH)D] < 50 nmol/L) were recruited. Participants were randomly allocated to receive either vitamin D_3_ (4000 IU/day) or matching placebo for 24 weeks. Between weeks 12 and 24, all participants completed multi-modal exercise three days per week while continuing with vitamin D/placebo. Mean changes in physical function (primary outcome: gait speed), body composition and biochemical parameters at weeks 12 and 24 were compared between groups.

**Results:**

Vitamin D supplementation, with or without exercise, had no effect on gait speed. From baseline to week 12, vitamin D supplementation increased serum 25(OH)D levels (placebo: 2.5 ± 14.7 nmol/L; treatment: 43.4 ± 18.4 nmol/L; *P* < 0.001) and reduced stair climb times (placebo: 0.3 ± 1.0 s; treatment: − 0.2 ± 1.0 s; *P* = 0.046). From 12 to 24 weeks, vitamin D supplementation combined with exercise decreased waist circumference (placebo: 1.3 ± 7.3 cm; treatment: − 3.0 ± 6.1 cm; *P* = 0.02) and waist-to-hip ratio (placebo: 0.01 ± 0.05; treatment: − 0.03 ± 0.05; *P* = 0.01) relative to placebo. Vitamin D supplementation, with or without exercise, had no effect on other physical function, body composition or metabolic health outcomes.

**Conclusion:**

Vitamin D supplementation had no effect on most physical function, body composition or metabolic health parameters when taken alone, or during exercise, in overweight or obese older adults with vitamin D deficiency. Vitamin D-related improvements in stair climb times and waist circumference suggest that future trials should explore the effects of vitamin D on muscle power, and its effects on body composition when combined with exercise, in populations with moderate or severe vitamin D deficiency.

**Supplementary Information:**

The online version contains supplementary material available at 10.1007/s00394-022-03038-z.

## Introduction

Overweight and obese older adults are at increased risk for vitamin D deficiency, which is associated with poor metabolic and musculoskeletal health, unfavorable body composition, and attenuated responses to exercise [1–5]. However, studies investigating the effects of vitamin D supplementation on these outcomes have been inconsistent, which is likely attributable to differing study durations, dosing regimens and baseline vitamin D status of participants [6, 7]. The greatest benefits following vitamin D supplementation appear to be in those with vitamin D deficiency [8–10].

In a study of 80 frail Japanese older adults, the greatest physical performance improvements following a three-month training program (exercises targeting balance, mobility and improvements in ability to complete activities of daily living) were observed in participants with baseline 25-hydroxyvitamin D [25(OH)D] levels > 67.5 nmol/L, with no improvements in those with 25(OH)D levels < 47.5 nmol/L, suggesting that higher vitamin D levels might enhance the beneficial effects of exercise [2]. Similarly, a meta-analysis by Stockton et al. reported that vitamin D supplementation increased lower-limb muscle strength, but only in individuals with moderate vitamin D deficiency [25(OH)D < 25 nmol/L] [8]. Few randomized controlled trials (RCTs) have investigated whether vitamin D can enhance exercise-related improvements in metabolic health, physical function and body composition. A recent meta-analysis in older adults demonstrated vitamin D supplementation combined with resistance training led to greater lower-limb muscle strength improvements compared with placebo plus exercise; however, only one of the three included studies recruited vitamin D-deficient individuals [11]. Since this meta-analysis, only two studies have investigated whether vitamin D supplementation enhances exercise responsiveness in vitamin D-deficient populations. In the first study, the authors reported that vitamin D supplementation (50,000 IU taken fortnightly for 3 months) in 52 adults with type 2 diabetes and vitamin D deficiency aged 40–65 years [body mass index (BMI) 27.7–29 kg/m^2^] led to greater decreases in insulin resistance following 12 weeks of resistance training compared with placebo [homeostatic model assessment for insulin resistance (HOMA-IR) change: − 48 vs. − 22%, respectively] [12]. However, the authors made no direct comparisons between these two groups, and mean baseline HOMA-IR values differed between groups by approximately 20%, so it is unclear whether these changes were statistically different [12]. In the second study, resistance training-induced gains in muscle mass and strength were not enhanced by 12 weeks of vitamin D supplementation (8000 IU/day) in 39 normal weight (BMI: 23.7 ± 2.5 kg/m^2^) vitamin D-deficient young men (age: 23.7 ± 2.5 years) [13]. Given that the beneficial effects of vitamin D supplementation might be more pronounced in older adults [9], and older adults with obesity are more likely to have physical performance limitations such as slow gait speed, a strong predictor of adverse health outcomes such as falls, fracture and mortality [14], it is important to determine whether correction of vitamin D deficiency enhances the functional benefits of exercise in this population. This might occur through several purported mechanisms including improvements in muscle protein synthesis, skeletal muscle energy metabolism, calcium homeostasis and fat oxidation, which have been reported following vitamin D supplementation [15–20].

Our objective was to determine whether, compared with placebo, vitamin D supplementation (4000 IU/day) taken prior to and during a 12-week exercise program improves physical function, body composition or metabolic health, in overweight or obese older adults with vitamin D deficiency.

## Methods

This 24-week parallel-group, double-blind, placebo-controlled pilot RCT included 50 overweight or obese (BMI ≥ 25 kg/m^2^ for persons of Caucasian, Hispanic or African origin and ≥ 23 kg/m^2^ for persons of Asian origin) [21] older adults aged 50–80 years with vitamin D deficiency [25(OH)D < 50 nmol/L]. Exclusion criteria included self-reported inability to walk 400 m non-stop unassisted (i.e., without the use of walking aids); inability to speak English; vitamin D supplementation ≥ 1000 IU/day; ≥ 4 weeks self-reported participation in a supervised exercise program targeted at weight loss or strength gains in the past six months; planning to be away from home for ≥ 2 weeks during the training phase; pregnancy or trying to become pregnant and self-reported diagnosis of: progressive neurological disorders, severe knee or hip osteoarthritis (awaiting a joint replacement), lung disease requiring regular use of supplemental oxygen, renal disease requiring dialysis or any other disorder of such severity that life expectancy was less than 12 months. Participants were also excluded if they had a stroke, hip or knee replacement, spinal surgery, myocardial infarction or major heart surgery in the past 6 months, or used medications contraindicated with vitamin D supplementation. Participants were recruited over three years from the local community in Melbourne, Australia, via print and online advertising. This RCT was conducted at a single site (Monash Health Translational Research Facility, Melbourne, Australia), conformed to CONSORT guidelines and was conducted according to the principles of the Declaration of Helsinki and approved by the Monash Health Human Research Ethics Committee (Protocol ID: HREC/15/MonH/182). Additionally, this trial was registered in the Australian New Zealand Clinical Trails Registry (ACTRN12616000563460). All participants provided written informed consent.

### Intervention and randomization

This 24-week intervention was divided into two 12-week phases: a pre-training phase, involving commencement of vitamin D supplementation or identical oral placebo, and a training phase, in which all participants continued to consume vitamin D or placebo whilst completing a multi-modal exercise program with weekly supervised sessions. There is debate surrounding effective and safe dosages of vitamin D_3_. Doses in the range of 400–1000 IU/day appear to have little or no effect on physical function in older adults [22] and the Institute of Medicine (IOM) states that the upper limit of vitamin D supplementation with minimal risk of adverse events is 4000 IU/day for most adults [23]. In a previous trial, we demonstrated that most participants with low 25(OH)D levels and risk factors for type 2 diabetes mellitus required doses of 4000 IU/day to achieve optimal 25(OH)D levels of 75 nmol/L over 6 months [24]. Daily vitamin D_3_ dosing also leads to greater increases in 25(OH)D levels compared with weekly and monthly dosing in older adults [25]. Therefore, for this 24-week trial in vitamin D-deficient overweight or obese older adults, eligible participants were randomized to either 4000 IU/day of oral vitamin D_3_ (Biological Therapies^®^ and Slade Pharmaceuticals), or identical placebo, using computer-generated randomization completed by an independent statistician (randomization sequence was inaccessible to study staff involved in outcome assessments and delivery of interventions). However, due to a requirement of our Human Research Ethics Committee, participants with 25(OH)D levels < 25 nmol/L were considered to have moderate vitamin D deficiency and were automatically allocated to the treatment group and prescribed vitamin D_3_, as per clinical recommendations [26–28].

### Pre-training phase

Participants were given an unmarked, sealed container with a 12-week supply of either vitamin D or placebo capsules after baseline and week 12 appointments. Participants were instructed to consume one capsule per day until their next clinic appointment. All capsules were identical and tasteless and both participants and study staff were blinded to treatment allocations.

### Training phase

Following week 12 measurements, all participants commenced an identical community-based, multi-modal exercise program, while adhering to the same supplementation protocol (i.e., receiving vitamin D or placebo) as per the pre-training phase. The exercise intervention followed the Lifestyle Interventions and Independence for Elders Study (LIFE) protocol [29]. LIFE is a progressive multi-modal exercise program that includes aerobic, resistance and balance exercise that has demonstrated effectiveness for reducing mobility limitations in older adults over a 2.6-year period [30]. The training phase consisted of one clinic-based (supervised) exercise session and two home-based exercise sessions per week. Supervised sessions were used to individually tailor and progressively increase exercise goals and identify/resolve barriers to exercise (e.g., injury/time). Participants progressed to three 75-min training sessions each week over 12 weeks. Each session consisted of a warm-up (slow walking and stretching), followed by aerobic training (moderate-intensity walking or jogging) and resistance training (leg-strengthening exercises included knee extension and flexion, squats, hip adduction and abduction and calf raises) and a cool-down (slow walking and stretches) [29]. Participants were instructed to complete aerobic and resistance training exercises at moderate intensity based on self-perceived exertion reported on the Borg scale [31]. All participants were provided with adjustable ankle weights to complete resistance training during clinic- and home-based exercise sessions. Following completion of the final exercise session in week 12, participants completed 24-week measurements.

### Questionnaires

Participants filled out questionnaires about general demographic information, chronic health conditions and their weekly physical activity levels. The International Physical Activity Questionnaire for the Elderly (IPAQ-E) was used to calculate the total days where participants performed more than 20 min of moderate or vigorous physical activity (MVPA) [32].

### Blood biochemistry

Blood samples were collected via venepuncture by a trained phlebotomist following an overnight fast of at least 10 h, and analyzed by a commercial pathology service (Monash Health Pathology) Serum 25(OH)D was analyzed using a direct competitive chemiluminescent immunoassay (DiaSorin Inc., USA; cat no: 310600) with level 1 and 2 control coefficients of variation (CV) of 10.7% and 6.5%, respectively (control kit: 310,601). Triglycerides (cat no: 445850) and high-density lipoprotein (HDL) cholesterol (cat no: 650207) were measured using commercial enzymatic assays (Beckman Coulter Inc., USA); triglycerides CV: 4.7 – 7.4% (BIORAD Liquichek control kits: 691 and 692) and HDL CV: 4.4 – 4.5% (BIORAD Liquichek control kits: 691 and 692). Low-density lipoprotein (LDL) concentrations were calculated using the following formula: LDL cholesterol = total cholesterol−HDL−(triglycerides/5) [33]. Serum fasting glucose was measured using a commercial enzymatic kit (Beckman Coulter Inc., USA; cat no: 472500) with a CV of 1.6–2.6% (BIORAD Liquichek control kits: 691 and 692) and insulin was measured using a radioimmunoassay (Beckman Coulter Inc., USA; cat no: 33410 and 33,415) with a CV of 4.6–6.9% (BIORAD Immunoassay Plus levels 1–3 (lyophilized) cat no: 370). HOMA-IR was calculated using the following formula: fasting insulin concentration x fasting glucose concentration/22.5.

### Anthropometry

Weight (Seca 804 electronic scales, Seca, Hamburg, Germany) and height (Seca 213 wall-mounted stadiometer, Seca, Hamburg, Germany) were measured with footwear and heavy items of clothing removed. BMI was calculated as weight in kilograms (kg) divided by height in meters squared (m^2^).

Waist and hip circumference were measured twice to the nearest 0.1 cm using a measuring tape (Seca 203). Waist circumference was measured in the mid-axillary plane at the midpoint between the inferior margin of the last rib and the crest of the ilium. Hip circumference was measured at the level of the greatest posterior protuberance of the buttocks. A third measurement was taken if the difference between measures one and two was greater than 2 cm. The average of these readings was then calculated. Waist-to-hip ratio (WHR) was calculated by dividing waist circumference by hip circumference.

### Physical function

Hand grip strength of the dominant hand was measured using a Jamar Plus Digital hydraulic hand grip dynamometer (Patterson Medical, Bolingbrook, IL, USA) [34]. Participants sat with their arms fully extended at shoulder height, parallel to the ground, and gripped the dynamometer with maximum force for three seconds. The test was repeated three times in the dominant arm with a 60 s rest between trials and the mean of the second two trials was used to calculate average hand grip strength (kg). The average stair climb time was calculated by timing two attempts at ascending a 10-step flight of stairs as quickly as possible (participants could use the handrail if required) [35]. Participants also completed a 400 m walk to objectively measure exercise capacity [36]. Participants were instructed to walk 400 m as quickly as possible without running in a hallway on a 20 m course (20 laps) and the total time taken was recorded in seconds.

Participants completed a short physical performance battery (SPPB), which is a validated measure of physical performance and disability in older adults [37]. Performance in three tasks (repeated chair stands, standing balance assessments and gait speed over a 2.44 m course) was used to calculate a summary score from 0 to 12 (higher score indicates better function). Participants completing repeated chair stands were instructed to cross their arms and attempt to stand up straight from a seated position five times as quickly as possible, without stopping in between repetitions. The timer was stopped once the participant completed five repetitions. Tests were terminated if participants used their arms to assist with standing, failed to come to a complete standing position, or if one minute had lapsed. After completion of the chair stand test, a score of 0–4 was given based on the time taken to complete five chair stands. Gait speed was measured using a 2.44 m course and a score of 0–4 was assigned based on the time it took to walk this distance at a normal walking speed. Balance was measured by asking participants to complete a semi-tandem stand with the heel of one foot placed beside the big toe of the opposite foot for 10 s. Those who could stand in the semi-tandem position for 10 s were then instructed to stand in a full-tandem position with their preferred foot in front of the other and the back of the heel on the front foot touching the toes of the back foot. Individuals who unable to hold the semi-tandem stand for 10 s were then instructed to stand with both feet side-by-side stand for 10 s. Following completion of the balance tests, a score of 0–4 was assigned based on performance in these tests.

### Body composition

Whole-body dual-energy X-ray absorptiometry (DXA) (Hologic Discovery A, Hologic, USA) was used to estimate whole-body fat and lean mass, as well as body fat percentage and visceral fat area (cm^2^). The sum of lean mass in the upper and lower limbs was used to calculate appendicular lean mass (ALM). ALM corrected for height was calculated as ALM divided by height (m^2^). The following equation was used to calculate upper-limb relative strength: average dominant arm hand grip strength (kg) divided by lean mass (determined by DXA) in the corresponding limb (kg) [38]. The DXA scanner was calibrated daily with the manufacturer’s spine phantom and the short-term intra-individual CV fat mass and ALM was 2.67% and 1.60%, respectively. All DXA scans were analyzed by one individual (JM).

A single 2.5 mm transverse peripheral quantitative computed tomography (pQCT) scan (Stratec XCT3000, Stratec Medizintechnik GmbH, Pforzheim, Germany) at a speed of 20 mm/s and voxel size of 0.8 mm was obtained at 66% of the tibial length of the non-dominant leg. The distance between the prominence of the medial malleolus and the tibial plateau was measured to determine the length of the tibia. To locate scan sites, a planar scout view of the distal tibia was used and reference lines were placed parallel to the distal joint surface of the tibia. Calf muscle density (mg/cm^3^) (density of tissue within the muscle compartment after removal of subcutaneous fat and bone) was estimated using manufacturer’s algorithms and software (version 6.2). Specifically, scans were analyzed using a smoothing filter (F03F05) at a threshold of 41 mg/cm^3^. The device was calibrated daily using the manufacturer’s phantom and the short-term intra-individual CV for muscle density was 1.2%. All pQCT scans were analyzed by one individual (JM).

### Intervention adherence and adverse events

Participants were asked to complete a physical activity diary (for prescribed exercises only) during each week of the training phase to determine adherence to the exercise protocol. Diaries from the previous week were returned to the study center in subsequent supervised exercise sessions. At 12- and 24-week appointments, participants returned any unused tablets, which were counted to estimate adherence to the supplementation protocol.

Information about adverse events was collected during testing sessions and weekly exercise sessions. Any untoward medical occurrence in participants that required some form of treatment was considered an adverse event, irrespective of whether it was thought to be related to the interventions.

### Study power

A priori sample size calculations were based on a clinically meaningful change in gait speed, based on findings from a previous study [39]. Gait speed was chosen as the primary physical function outcome because slow gait speed is associated with adverse clinical outcomes [14], not limited by ceiling effects, and responds to exercise targeting the lower limbs [30]. Adjusting for a loss to follow-up of 20% (*n* = 10), this study had more than 80% power to detect a clinically meaningful 0.10 m/s (SD 0.12 m/s) net difference in gait speed between vitamin D and placebo, at an alpha level of 5% [39].

### Statistical analysis

Our main analysis followed intention-to-treat principles and missing data were imputed using multiple imputation. Multiple imputation models included age, sex, BMI at baseline and all available values of each respective outcome at all time points. Twenty imputed datasets were created for all outcome measures and pooled results were analyzed. Per-protocol analyses were also performed in participants who achieved at least 66% adherence to the exercise intervention (equivalent to 2 sessions per week) and 80% adherence to the vitamin D supplementation protocol.

Continuous data were assessed for normality using boxplots and Shapiro–Wilk tests. 12- and 24-week changes in all outcomes and group by time interactions were analyzed using generalized linear mixed models. We also performed subgroup analyses in women, men and overweight or obese participants. Thresholds for overweight were BMI ≥ 25 and < 30 kg/m^2^ for persons of Caucasian, Hispanic or African origin and ≥ 23 and 25 < kg/m^2^ for persons of Asian origin. Thresholds for obesity were BMI ≥ 30 kg/m^2^ for persons of Caucasian, Hispanic or African origin and ≥ 25 kg/m^2^ for persons of Asian origin. *P* values < 0.05 or 95% confidence intervals (CI) not including the null point were considered statistically significant. All analyses were performed in SPSS Statistics version 25 (IBM, Armonk, NY, USA).

## Results

The cohort had a mean age of 60 ± 6 years, BMI of 30.6 ± 5.7 kg/m^2^ and baseline serum 25(OH)D levels of 41 ± 10 nmol/L. Nine participants (18%) had baseline 25(OH)D levels < 30 nmol/L and 31 (62%) were women. Both groups had similar ages, levels of physical activity and proportions of individuals that were previous or current smokers (Table 1). The proportion of participants recruited in summer, autumn, winter and spring was also similar between groups.

**Table 1:** Descriptive characteristics

	Placebo *N* = 24	Vitamin D *N* = 26
Age (years)	60 ± 6.2	59.6 ± 6.7
Women (%)	15 (62.5)	16 (61.5)
Current or previous smoker (%)	11 (46)	9 (35)
Diabetes (%)	2 (8.3)	5 (19.2)
Osteoporosis (%)	2 (8.3)	1 (3.8)
Osteoarthritis (%)	6 (25)	3 (11.5)
Self-reported moderate to vigorous physical activity (days/week)	3 (2, 7)	3 (1, 5)
Season (%)		
Summer	4 (16.7)	5 (19.2)
Autumn	4 (16.7)	6 (23.1)
Winter	10 (41.7)	8 (30.8)
Spring	6 (25)	7 (26.9)

Data are presented as mean ± SD or median (IQR)

**Fig. 1:**
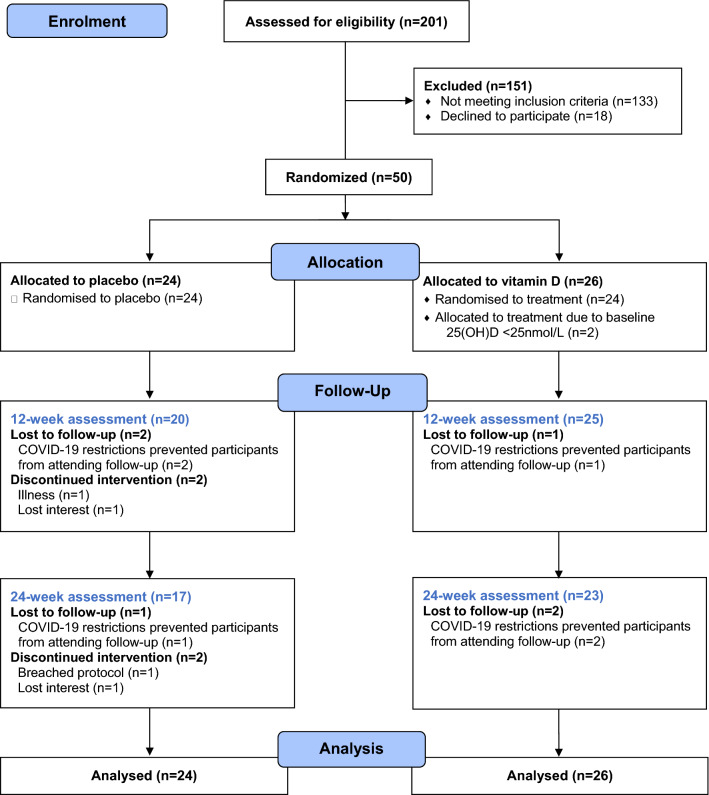
Study design and flow of participants

Five participants (vitamin D *n* = 1; placebo *n* = 4) did not complete the 12-week follow-up and five participants (vitamin D *n* = 2; placebo *n* = 3) did not complete the 24-week follow-up (Fig. 1). The main reason for attrition in both groups was the implementation of restrictions on research during COVID-19 pandemic in 2020 (*n* = *6*). Other reasons for attrition included illness (*n* = 1), loss of interest (*n* = 2) and breaching protocol (commenced over-the-counter vitamin D supplementation; *n* = 1). Mean (and median) adherence to the supplementation protocol at 12 and 24 weeks in the placebo group was 96% (100%) and 97% (100%), respectively. Mean (and median) adherence to the supplementation protocol at 12 and 24 weeks in the vitamin D group was 95% (100%) and 94% (100%), respectively. Mean (and median) exercise adherence between weeks 12 and 24 was 67% (83%) in the placebo group and 71% (83%) in the vitamin D group.

There were no supplement-related adverse events reported in this study. A total of 3/50 participants (vitamin D *n* = 1; placebo *n* = 2) reported musculoskeletal complaints during the exercise phase. One participant reported hip abductor and adductor soreness, one participant reported sore and swollen ankles and one participant reported soreness along the left lateral thigh. The participant who reported lateral thigh soreness consulted with a medical professional as this was related to a pre-existing injury. All participants applied conservative management that usually involved icing and/or the consumption of over-the-counter analgesics. All participants who reported musculoskeletal complaints continued exercising with modified programs.

**Fig. 2:**
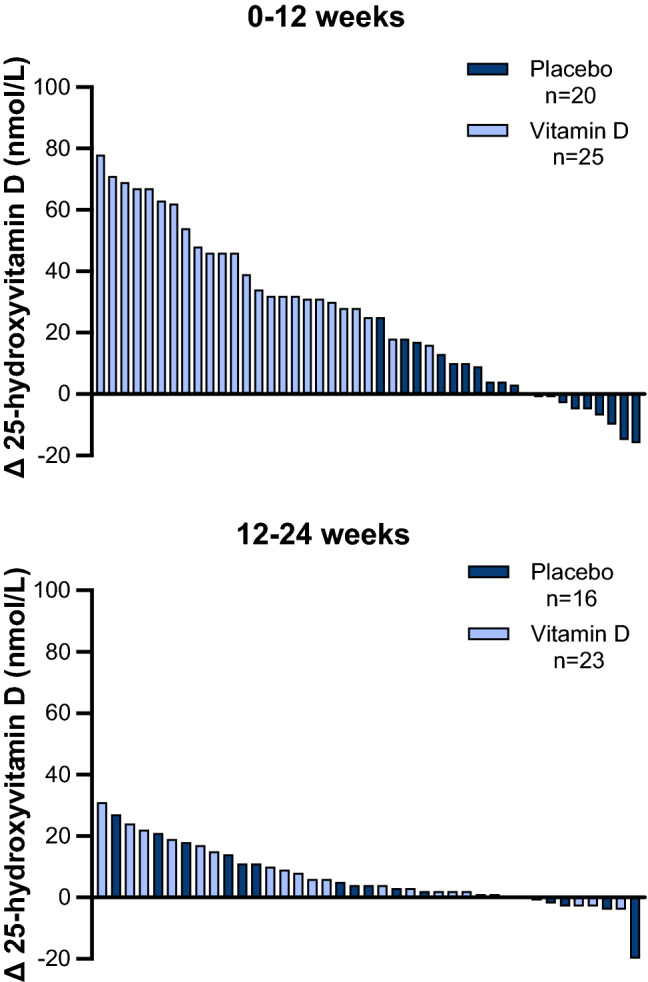
Twelve- and 24-week changes in 25-hydroxyvitamin D in individual participants

Between baseline and week 12, serum 25(OH)D levels increased in all vitamin D group participants (range: 16–78 nmol/L) and 32 (64%) of individuals achieved 25(OH)D levels ≥ 75 nmol/L (Fig. 2). In the placebo group, half of the participants had small increases in 25(OH)D levels, while the other half of participants had no changes or decreases (range: − 16 to 25 nmol/L). Between 12 and 24 weeks, most (74%) participants in the vitamin D group still had increases in 25(OH)D, but the magnitude of these increases was smaller compared with those at week 12 (range: − 4 to 31 nmol/L). Despite the smaller increases in 25(OH)D in the vitamin D group, 78% of participants had 25(OH)D levels ≥ 75 nmol/L at 24 weeks. Most participants in the placebo group also had small increases in 25(OH)D levels (range: − 21 to 27 nmol/L) at 24 weeks, but no participants had 25(OH)D levels above 75 nmol/L at week 12 or 24.

**Fig. 3:**
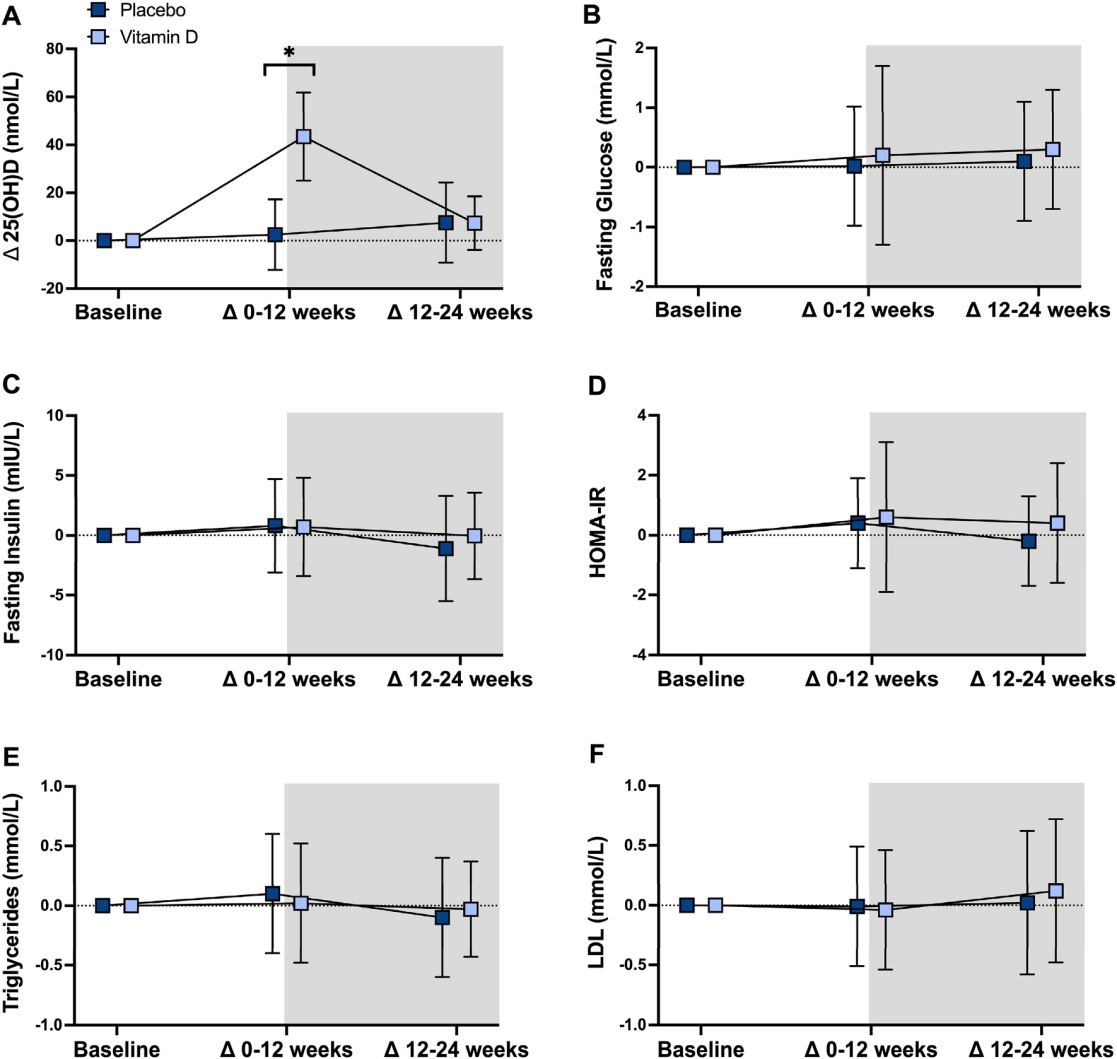
Twelve- and 24-week changes in biochemical parameters in vitamin D and placebo. Data are mean ± SD. Between-group differences analyzed using linear mixed models. 25(OH)D — 25-hydroxyvitamin D; *HOMA-IR* homeostatic model assessment of insulin resistance, *LDL* low-density lipoprotein. Pre-training phase = unshaded; training phase = shaded. Placebo: *n* = 24; Vitamin D: *n* = 26

**Fig. 4:**
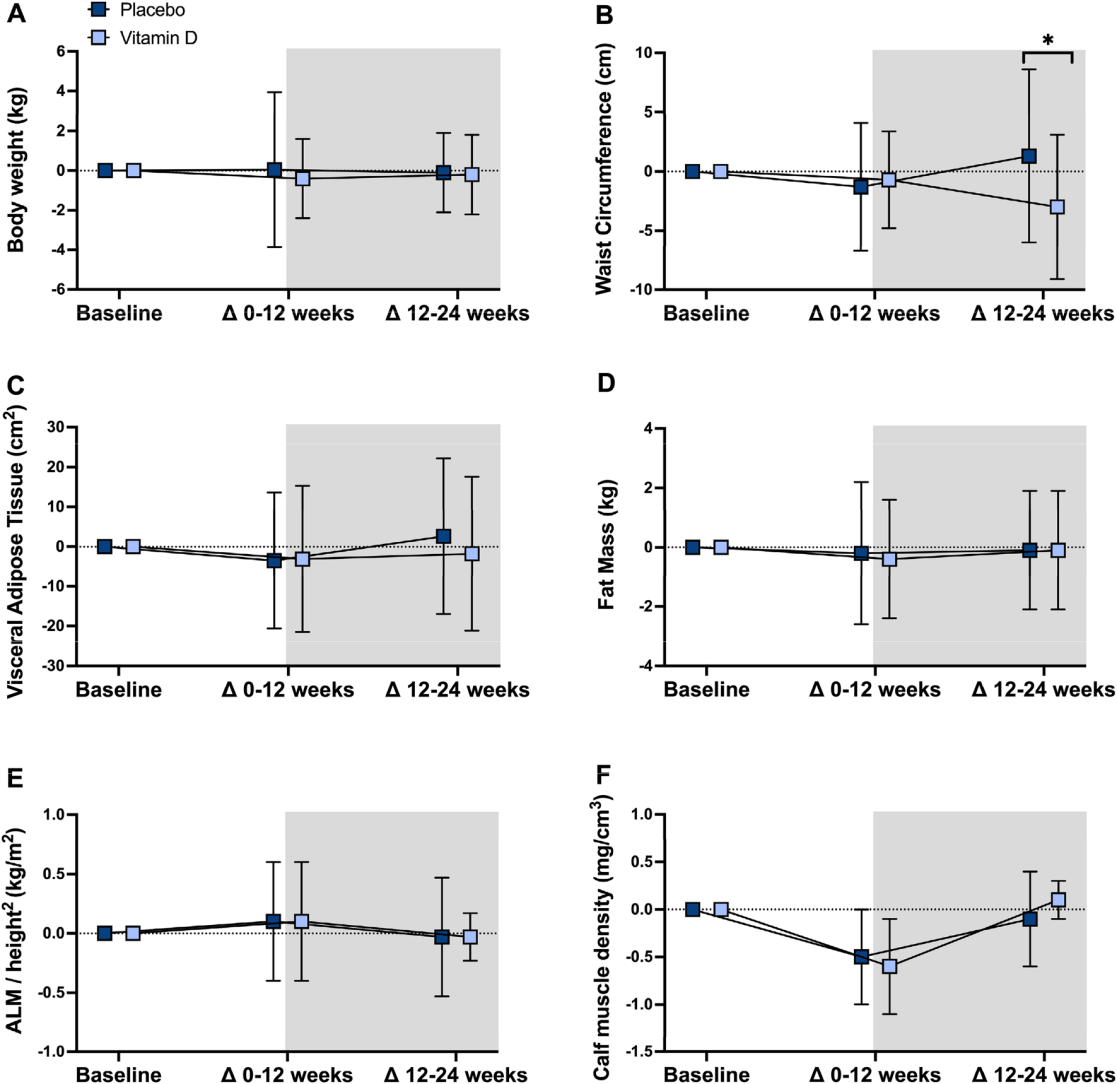
Twelve- and 24-week body composition changes in vitamin D and placebo. Data are mean ± SD. Between-group differences analyzed using linear mixed models. *P < 0.05; *ALM/H*^*2*^ appendicular lean mass/height^2^. Pre-training phase = unshaded; training phase = shaded. Placebo: *n* = 24; Vitamin D: *n* = 26

**Fig. 5:**
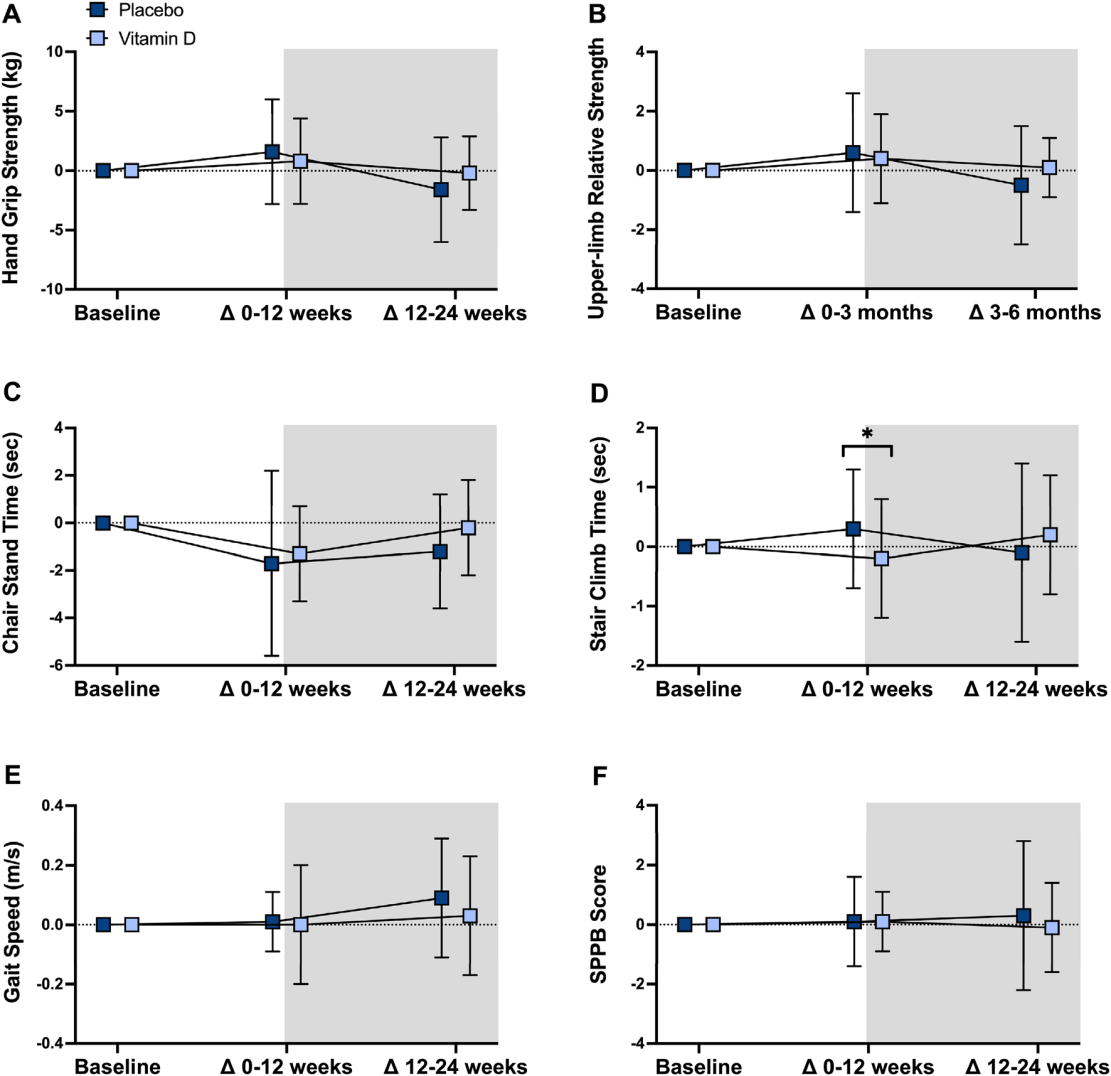
Twelve- and 24-week body composition changes in vitamin D and placebo. Data are mean ± SD. Between-group differences analyzed using linear mixed models. *P < 0.05; *SPPB* short physical performance battery. Pre-training phase = unshaded; training phase = shaded. Placebo: *n* = 24; Vitamin D: *n* = 26

Supplementary Table S1 presents mean baseline values and intention-to-treat analyses comparing changes in blood biochemistry, body composition and physical function after 12 and 24 weeks in the placebo and vitamin D groups. Changes in most of these outcome measures are also graphed in Figs. 3, 4, 5. The vitamin D group had significant increases in 25(OH)D levels relative to placebo from baseline to 12 weeks, but not from 12 to 24 weeks. Waist circumference and WHR decreased from 12 to 24 weeks, and stair climb time decreased from baseline to 12 weeks, in the vitamin D group compared with placebo. The significant decrease in waist circumference (net difference: − 4.4 cm [95%CI − 8.1, − 0.8 cm], *P* = 0.017) and WHR (net difference: − 0.1 [95%CI − 0.1, − 0.02], *P* = 0.001) in the vitamin D group compared with placebo from 12 to 24 weeks remained significant after adjusting for change in fat mass. All findings were similar after adjusting for season, excluding the two participants with baseline 25(OH)D levels below 25 nmol/L, and performing complete case analyses (data not shown).

Per-protocol analyses were also performed in 23 participants (vitamin D *n* = 13; placebo *n* = 10) who achieved at least 66% adherence to the exercise intervention (equivalent to 2 sessions per week) and at least 80% adherence to the vitamin D supplementation protocol (Supplementary Table S2). All results were unchanged except the decreases in waist circumference (12–24 weeks) and stair climb time (baseline to 12 weeks) in the vitamin D group relative to placebo became non-significant.

We performed exploratory sex- and obesity-stratified subgroup analyses (Supplementary Tables S3–S6). In all subgroups, between-group differences in 25(OH)D changes were similar to our main analysis at both time points. In women, men, and participants with obesity, waist circumference and/or WHR decreases were observed in the vitamin D group compared with placebo from 12 to 24 weeks. In women and overweight participants, hand grip strength and upper-limb muscle quality decreased in the placebo group relative to the vitamin D group from 12 to 24 weeks. In men, calf muscle density increased in the vitamin D group relative to placebo from 12 to 24 weeks. In overweight participants, the vitamin D group also lost fat mass relative to placebo from 12 to 24 weeks.

## Discussion

This 24-week RCT demonstrated that 4000 IU/day vitamin D supplementation had no effect on gait speed when taken with or without exercise. Following multi-modal exercise, vitamin D supplementation decreased waist circumference compared with placebo in overweight or obese older adults with vitamin D deficiency. This finding may be clinically relevant as vitamin D supplementation is a safe, low-cost addition to formal exercise programs, especially in vitamin D-deficient individuals. Vitamin D supplementation taken alone also reduced stair climb times; however, it had no beneficial effects on other biochemical, body composition or physical function parameters when taken alone or during exercise.

Increases in serum 25(OH)D levels following high-dose vitamin D supplementation have been well-documented in overweight or obese populations with vitamin D deficiency [40–42]. In our study, a dosing regimen of 4000 IU/day was sufficient to increase serum 25(OH)D levels from < 50 to ≥ 75 nmol/L in 64% and 78% of participants in the vitamin D group after 12 and 24 weeks, respectively. Adherence to both vitamin D and placebo was very high (> 95%) and at the abovementioned doses, there were no supplement-related adverse events reported in this study. The magnitude of vitamin D change was blunted in those taking vitamin D between weeks 12–24, which was due to 25(OH)D levels stabilizing over this period.

Following multi-modal exercise, vitamin D supplementation decreased waist circumference compared with placebo, which also led to greater WHR decreases. This finding was also observed in most of our subgroup analyses. Improvements in WHR following high-dose vitamin D_3_ supplementation have been reported in overweight or obese Asians with vitamin D deficiency [43], and higher 25(OH)D levels have been associated with greater waist circumference losses in overweight or obese young adults following a 12-week resistance training intervention [44]. Waist circumference losses following multi-modal exercise in the vitamin D group might be explained by individuals with higher or replete 25(OH)D levels experiencing increased fat oxidation during exercise. Ellis et al. showed that serum 25(OH)D levels were inversely associated with respiratory quotients (lower values indicate greater fat oxidation) measured during several submaximal exercise tests in postmenopausal women [20]. It is unclear why waist circumference and WHR decreased independently of total body fat mass changes in response to combined vitamin D plus exercise, although the Framingham Heart Study showed that visceral fat mass has stronger associations with 25(OH)D levels than subcutaneous fat [45]. The European Prospective Investigation into Cancer and Nutrition (EPIC) study demonstrated that higher abdominal adiposity measured by waist circumference or WHR is related to increased mortality risk, independent of BMI [46]. We observed a 4.3 cm net difference in waist circumferences changes between groups between 12 and 24 weeks, and the EPIC study reported that a 5 cm greater waist circumference was associated with significantly increased mortality risk at a given BMI in men and women (17% and 13%, respectively) [46]. In the current study, between-group differences in DXA-determined visceral adipose tissue changes were supportive of our waist circumference findings; however, they were not statistically significant. This could be related to the decrease in precision of visceral adipose tissue estimates with increasing BMI categories [47], or insufficient statistical power.

Vitamin D supplementation taken alone and in combination with exercise had no beneficial effects on muscle density. Our sub-analysis in men showed that vitamin D supplementation increased muscle density during exercise, however, this finding should be interpreted with caution given the low numbers in our subgroups. Muscle density is an indirect measure of inter- and intramuscular adipose tissue (IMAT), and lower muscle density is associated with poor mobility [48] and increased falls risk in older adults [49]. Studies suggest that having high amounts of IMAT can blunt exercise responsiveness in older adults [50, 51]. Nevertheless, several studies have also demonstrated that exercise can decrease IMAT [50, 52, 53]; however, very few have explored whether vitamin D supplementation augments the beneficial effects of exercise on this ectopic fat depot. Interestingly, there appears to be dose-dependent effects of calcitriol, the active form of vitamin D, on transdifferentiation of myogenic precursor cells into adipose cells [54]. Data also show that vitamin D supplementation influences myostatin and insulin-like growth factor 1, which are important regulators of both muscle mass and adipogenesis [55, 56]. In a similar study to ours, Miller et al. reported that muscle density changes were not enhanced by daily vitamin D and protein supplementation (2000 IU vitamin D_3_; 20 g whey protein on non-training days and 40 g on training days) during progressive resistance training in vitamin D replete overweight or obese middle-aged and older adults with type 2 diabetes [57]. On the contrary, Englund et al. reported that daily vitamin D and protein supplementation (800 IU vitamin D_3_; 20 g whey protein) augmented IMAT losses following a multi-modal exercise program in vitamin D insufficient [baseline 25(OH)D = 9–24 ng/mL] older adults with mobility limitations [53]. Interestingly, this study also demonstrated that vitamin D and protein supplementation during exercise led to an increase in muscle density in normal-density muscle, but not in low-density muscle [53]. Therefore, it is possible that vitamin D supplementation had very little effect on muscle density/IMAT in our study and in Miller et al. [57] because both populations were overweight or obese, which likely led to a high proportion of participants having “supplement resistant” low-density muscle. Further research exploring thresholds for normal- and low-density muscle, and how they are influenced by different exercise and nutritional interventions in older adults with obesity, are warranted.

Stair climb times improved in response to vitamin D supplementation alone, but not in combination with exercise. We previously reported higher 25(OH)D levels are associated with better stair climb times in overweight or obese older women, but not men [58]. It is possible that vitamin D supplementation increases muscle power, the component of physical function assessed via stair climb tests, but not general physical performance outcomes such as gait speed (our primary outcome), which did not significantly change in this study. Vitamin D supplementation has been shown to increase muscle power in pre-frail community-dwelling older adults [59]. This could occur due to increases in type II muscle fiber size and percentage, which have also been reported to increase in response to vitamin D supplementation [56, 60]. However, we note that the confidence intervals for between-group differences in stair climb time from baseline to 12-week approach zero, so this finding should also be interpreted with caution. Nevertheless, future studies in this population should include direct assessments of muscle power via techniques such as jumping mechanography [61–63], and/or direct measures of muscle fiber composition via muscle biopsies.

The LIFE study we based our exercise intervention on reported reduced risk for disability in an at-risk population of older adults (aged 70–89 years) with physical function limitations (inclusion criteria: SPPB scores ≤ 9 or inability to walk 400 m) following multi-modal exercise [30]. Poorer baseline physical function in the LIFE cohort likely explains the greater efficacy of their exercise intervention compared with ours; their cohort had mean SPPB scores of 7/12, whereas our cohort had mean SPPB scores of 11/12 [30]. Reductions in disability risk in the LIFE study were observed in participants with lower SPPB scores (< 8) and slower gait speed (< 0.8 m/s), but not in subgroups with better SPPB scores and gait speed at baseline [30]. The LIFE study also reported that for every one SD increase in baseline BMI, participants had 19% lower odds of achieving a change in gait speed ≥ 0.05 m/s following their intervention [64]. Therefore, future studies aiming to improve physical performance in overweight and obese older adults without any functional deficits may need to prescribe alternative multi-modal exercise interventions involving higher exercise intensities and/or skeletal muscle loading to illicit greater functional improvements.

Consistent with our findings, recent meta-analyses have showed that vitamin D supplementation alone had no beneficial effect on muscle strength, size and performance [65, 66]. Our findings are also similar to those of Savolainen et al. described earlier [13] and another recent study by Molmen et al. [67], who reported that vitamin D supplementation had no effect on resistance training-induced changes in muscle strength, mass and performance in older adults. Other recent exercise and vitamin D trials have also reported no additive effects of vitamin D supplementation on aerobic capacity when combined with aerobic [68] or resistance exercise [69].

Strengths of this study included a well-characterized overweight/obese population with low baseline serum 25(OH)D levels, a comprehensive battery of physical function tests, data on important potential confounders, as well as the successful delivery of an evidence-based, multi-modal exercise program designed to be undertaken in a home-based setting to increase translatability and implementation [29]. We also had high participant retention and adherence to the supplementation protocol (which was effective in achieving optimal 25(OH)D levels in almost all participants in the vitamin D group). Limitations of this study include the relatively small sample size and modest adherence to the exercise intervention in both groups. Our exercise intervention also had a modest effect on most outcomes assessed in this study, which is unlikely to be related to the duration of our exercise intervention (12 weeks) given similar studies have shown significant improvements within the same timeframe [13, 44, 56]. Instead, the modest effect of our exercise intervention was likely related to the limited skeletal muscle loading associated with the use of ankle weights for exercise progression (each ankle weight had a maximum weight of 5 kg). Baseline values of outcomes in our study were comparable to those of participants recruited in cross-sectional studies with similar inclusion criteria [48, 70], but it is possible that advertising our exercise and dietary intervention led to volunteer bias. Assessment of physical performance via the SPPB might have limited sensitivity due to ceiling effects in our relatively well-functioning population. We did not have precision data for some of our body composition and physical function outcomes, which may limit the reliability of our findings. Our inclusion of only overweight or obese adults means that while addressing the main target population who would benefit from vitamin D supplementation, our results might not be generalizable to other populations. Future studies would be improved by prescribing resistance exercises at higher loads and intensities to illicit greater exercise-related improvements in physical function, body composition and metabolic health, which may be required to enhance exercise responsiveness via vitamin D supplementation. Future studies would also be improved by assessing 25(OH)D using the gold-standard liquid chromatography–mass spectrometry method and targeting individuals with moderate and severe vitamin D deficiency.

In conclusion, vitamin D_3_ supplementation (4000 IU/day) had no effect on gait speed when taken with or without exercise. However, vitamin D_3_ supplementation is effective in achieving optimal serum 25(OH)D levels and decreased waist circumference following multi-modal exercise in overweight or obese older adults with vitamin D deficiency. Vitamin D supplementation taken alone also reduced stair climb times, however, it had no beneficial effects on any other biochemical, body composition or physical function parameters when taken alone, or in combination with exercise. Future trials should focus on populations with moderate or severe vitamin D deficiency as they are more likely to experience therapeutic benefits from vitamin D supplementation.

## Supplementary Information

Below is the link to the electronic supplementary material.

Supplementary file1 (DOCX 100 KB)
