# Supplementary material for: Vitamin D supplementation and exercise for improving physical function, body composition and metabolic health in overweight or obese older adults with vitamin D deficiency: a pilot randomized, double-blind, placebo-controlled trial
Source: Eur J Nutr. 2022 Nov 4;62(2):951–64. doi: 10.1007/s00394-022-03038-z (PMC9638202; doi:10.1007/s00394-022-03038-z)
Supplement: Supplementary file 1 — Supplementary file1 (DOCX 100 KB) [file 394_2022_3038_MOESM1_ESM.docx]

**Vitamin D Supplementation and Exercise for Improving Physical Function, Body Composition and Metabolic Health in Overweight or Obese Older Adults with Vitamin D Deficiency: A Pilot Randomized, Double-Blind, Placebo-Controlled Trial**

^1,2^Jakub Mesinovic (ORCID: 0000-0003-1297-2731**)**

^1,3^Alexander J Rodriguez (ORCID: 0000-0002-2955-0642)

^1^Mavil May Cervo

^1^Anoohya Gandham

^1^Cecilia LH Xu

^1,2^Costas Glavas

^1^Barbora de Courten

^1^Ayse Zengin

^1^Peter R Ebeling

^1,2^David Scott

^1^Department of Medicine, School of Clinical Sciences at Monash Health, Monash University, Clayton, Victoria, Australia.

^2^Deakin University, Geelong, Australia, Institute for Physical Activity and Nutrition (IPAN), School of Exercise and Nutrition Sciences

^3^School of Medical and Health Sciences, Edith Cowan University, Perth, WA, Australia.

**Corresponding author and address for reprints:**

Dr. Jakub Mesinovic

Deakin University, Geelong, Australia, Institute for Physical Activity and Nutrition (IPAN)

School of Exercise and Nutrition Sciences

E-mail: [jakub.mesinovic@deakin.edu.au](mailto:jakub.mesinovic@deakin.edu.au)

**Supplementary Data**

**Supplementary Table S1.** Mean baseline values and intention-to-treat analyses of between-group changes in blood biochemistry, body composition and physical function after 12 and 24 weeks in placebo and vitamin D groups.

|  | Placebo  N=24 | Vitamin D  N=26 | Net Difference (95%CI) | Group x Time  (P-value) |
| --- | --- | --- | --- | --- |
| **Blood biochemistry** |  |  |  |  |
| **25-hydroxyvitamin D (nmol/L)** |  |  |  |  |
| Baseline | 41.8 ± 9.8 | 39.5 ± 10.7 |  |  |
| ﻿Δ 0-12 weeks | 2.5 ± 14.7 | 43.4 ± 18.4 | **40.9 (31.7, 50.1)** | **<0.001** |
| Δ 12-24 weeks | 7.5 ± 16.7 | 7.3 ± 11.2 | -0.2 (-8.2, 7.7) | 0.95 |
| **Fasting glucose (mmol/L)** |  |  |  |  |
| Baseline | 5.5 ± 1.0 | 5.8 ± 1.5 |  |  |
| ﻿Δ 0-12 weeks | 0.02 ± 1.0 | 0.2 ± 1.5 | 0.2 (-0.5, 0.9) | 0.54 |
| Δ 12-24 weeks | 0.1 ± 1.0 | 0.3 ± 1.0 | 0.3 (-0.2, 0.7) | 0.21 |
| **Fasting insulin (mUL)** |  |  |  |  |
| Baseline | 9.5 ± 6.9 | 9.2 ± 5.6 |  |  |
| ﻿Δ 0-12 weeks | 0.8 ± 3.9 | 0.7 ± 4.1 | -0.1 (-2.5, 2.2) | 0.90 |
| Δ 12-24 weeks | -1.1 ± 4.4 | -0.04 ± 3.6 | 1.1 (-1.2, 3.3) | 0.35 |
| **HOMA-IR (units)** |  |  |  |  |
| Baseline | 2.4 ± 2.0 | 2.5 ± 1.5 |  |  |
| ﻿Δ 0-12 weeks | 0.4 ± 1.5 | 0.6 ± 2.5 | 0.2 (-0.9, 1.4) | 0.68 |
| Δ 12-24 weeks | -0.2 ± 1.5 | 0.4 ± 2.0 | 0.6 (-0.3, 1.5) | 0.17 |
| **Triglycerides (mmol/L)** |  |  |  |  |
| Baseline | 1.4 ± 1.0 | 1.4 ± 0.5 |  |  |
| ﻿Δ 0-12 weeks | 0.1 ± 0.5 | 0.02 ± 0.5 | -0.1 (-0.4, 0.2) | 0.68 |
| Δ 12-24 weeks | -0.1 ± 0.5 | -0.03 ± 0.4 | 0.1 (-0.1, 0.3) | 0.41 |
| **HDL (mmol/L)** |  |  |  |  |
| Baseline | 1.55 ± 0.4 | 1.52 ± 0.3 |  |  |
| ﻿Δ 0-12 weeks | -0.04 ± 0.2 | -0.06 ± 0.1 | -0.01 (-0.1, 0.1) | 0.81 |
| Δ 12-24 weeks | 0.07 ± 0.3 | 0.03 ± 0.2 | -0.03 (-0.2, 0.1) | 0.65 |
| **LDL (mmol/L)** |  |  |  |  |
| Baseline | 3.58 ± 0.8 | 3.25 ± 1.2 |  |  |
| ﻿Δ 0-12 weeks | -0.01 ± 0.5 | -0.04 ± 0.5 | -0.02 (-0.3, 0.3) | 0.89 |
| Δ 12-24 weeks | 0.02 ± 0.6 | 0.12 ± 0.6 | 0.1 (-0.2, 0.4) | 0.55 |
| **Body composition** |  |  |  |  |
| **Weight (kg)** |  |  |  |  |
| Baseline | 86.4 ± 17.1 | 83.7 ± 16.3 |  |  |
| ﻿Δ 0-12 weeks | 0.04 ± 3.9 | -0.4 ± 2.0 | -0.4 (-2.1, 1.3) | 0.67 |
| Δ 12-24 weeks | -0.1 ± 2.0 | -0.2 ± 2.0 | -0.03 (-1.2, 1.1) | 0.97 |
| **BMI (kg/m^2^)** |  |  |  |  |
| Baseline | 31.5 ± 6.4 | 29.8 ± 4.6 |  |  |
| ﻿Δ 0-12 weeks | 0.1 ± 1.5 | -0.1 ± 1.0 | -0.2 (-0.9, 0.5) | 0.47 |
| Δ 12-24 weeks | 0.04 ± 1.0 | -0.02 ± 1.0 | -0.06 (-0.5, 0.4) | 0.81 |
| **Waist circumference (cm)** |  |  |  |  |
| Baseline | 103.9 ± 12.2 | 102.8 ± 12.2 |  |  |
| ﻿Δ 0-12 weeks | -1.3 ± 5.4 | -0.7 ± 4.1 | 0.7 (-2.0, 3.3) | 0.62 |
| Δ 12-24 weeks | 1.3 ± 7.3 | -3.0 ± 6.1 | **-4.3 (-7.9, -0.6)** | **0.02** |
| **Hip circumference (cm)** |  |  |  |  |
| Baseline | 113.0 ± 11.8 | 110.0 ± 12.2 |  |  |
| ﻿Δ 0-12 weeks | -0.1 ± 3.4 | -0.4 ± 3.1 | -0.3 (-2.1, 1.5) | 0.51 |
| Δ 12-24 weeks | -0.4 ± 3.9 | 0.8 ± 3.1 | 1.1 (-0.7, 3.0) | 0.23 |
| **Waist Hip Ratio** |  |  |  |  |
| Baseline | 0.92 ± 0.05 | 0.94 ± 0.05 |  |  |
| ﻿Δ 0-12 weeks | -0.01 ± 0.05 | -0.004 ± 0.01 | 0.01 (-0.02, 0.03) | 0.60 |
| Δ 12-24 weeks | 0.01 ± 0.05 | -0.03 ± 0.05 | **-0.04 (-0.1, -0.01)** | **0.01** |
| **ALM (kg)** |  |  |  |  |
| Baseline | 22.7 ± 5.9 | 22.3 ± 5.6 |  |  |
| ﻿Δ 0-12 weeks | 0.4 ± 1.5 | 0.1 ± 0.5 | -0.2 (-0.8, 0.4) | 0.47 |
| Δ 12-24 weeks | -0.1 ± 0.5 | -0.1 ± 0.5 | -0.04 (-0.4, 0.3) | 0.87 |
| **ALM/height^2^ (kg)** |  |  |  |  |
| Baseline | 8.2 ± 1.5 | 7.9 ± 1.5 |  |  |
| ﻿Δ 0-12 weeks | 0.1 ± 0.5 | 0.1 ± 0.5 | -0.1 (-0.3, 0.1) | 0.54 |
| Δ 12-24 weeks | -0.03 ± 0.5 | -0.03 ± 0.2 | 0.0 (-0.1, 0.1) | 0.98 |
| **Fat mass (kg)** |  |  |  |  |
| Baseline | 33.7 ± 11.8 | 31.8 ± 10.2 |  |  |
| ﻿Δ 0-12 weeks | -0.2 ± 2.4 | -0.4 ± 2.0 | -0.2 (-1.4, 0.9) | 0.76 |
| Δ 12-24 weeks | -0.1 ± 2.0 | -0.1 ± 2.0 | 0.02 (-1.0, 1.1) | 0.97 |
| **Body fat percentage** |  |  |  |  |
| Baseline | 40.4 ± 8.8 | 39.7 ± 8.2 |  |  |
| ﻿Δ 0-12 weeks | -0.4 ± 1.5 | -0.3 ± 1.5 | 0.1 (-0.8, 1.0) | 0.64 |
| Δ 12-24 weeks | 0.3 ± 1.5 | -0.1 ± 1.5 | -0.4 (-1.3, 0.5) | 0.39 |
| **Visceral adipose tissue (cm^2^)** |  |  |  |  |
| Baseline | 145.7 ± 57.8 | 145.1 ± 57.6 |  |  |
| ﻿Δ 0-12 weeks | -3.5 ± 17.1 | -3.1 ± 18.4 | 0.1 (-9.6, 9.8) | 0.61 |
| Δ 12-24 weeks | 2.6 ± 19.6 | -1.8 ± 19.4 | -3.7 (-14.4, 7.0) | 0.49 |
| **Calf muscle density (mg/cm^3^)** |  |  |  |  |
| Baseline | 74.3 ± 2.9 | 74.2 ± 2.5 |  |  |
| ﻿Δ 0-12 weeks | -0.5 ± 2.0 | -0.6 ± 2.0 | -0.1 (-1.2, 0.9) | 0.95 |
| Δ 12-24 weeks | -0.1 ± 2.4 | 0.1 ± 1.5 | 0.2 (-0.8, 1.1) | 0.77 |
| **Physical function** |  |  |  |  |
| **Hand grip strength (kg)** |  |  |  |  |
| Baseline | 32.1 ± 14.7 | 33.6 ± 11.7 |  |  |
| ﻿Δ 0-12 weeks | 1.6 ± 4.4 | 0.8 ± 3.6 | -0.8 (-2.9, 1.3) | 0.70 |
| Δ 12-24 weeks | -1.6 ± 4.4 | -0.2 ± 3.1 | 1.4 (-0.6, 3.4) | 0.19 |
| **Upper-limb relative strength** |  |  |  |  |
| Baseline | 11.0 ± 3.4 | 11.6 ± 3.1 |  |  |
| ﻿Δ 0-12 weeks | 0.6 ± 2.0 | 0.4 ± 1.5 | -0.2 (-1.1, 0.7) | 0.54 |
| Δ 12-24 weeks | -0.5 ± 2.0 | 0.1 ± 1.0 | 0.6 (-0.2, 1.4) | 0.11 |
| **Chair stand time (s)** |  |  |  |  |
| Baseline | 10.3 ± 5.4 | 9.2 ± 2.5 |  |  |
| ﻿Δ 0-12 weeks | -1.7 ± 3.9 | -1.3± 2.0 | 0.4 (-1.3, 2.0) | 0.66 |
| Δ 12-24 weeks | -1.2 ± 2.4 | -0.2 ± 2.0 | 1.0 (-0.2, 2.2) | 0.10 |
| **Stair climb time (s)** |  |  |  |  |
| Baseline | 4.6 ± 2.0 | 4.2 ± 1.5 |  |  |
| ﻿Δ 0-12 weeks | 0.3 ± 1.0 | -0.2 ± 1.0 | **-0.5 (-1.1, -0.01)** | **0.046** |
| Δ 12-24 weeks | -0.1 ± 1.5 | 0.2 ± 1.0 | 0.3 (-0.4, 1.0) | 0.39 |
| **Gait speed (m/s)** |  |  |  |  |
| Baseline | 0.85 ± 0.2 | 0.89 ± 0.2 |  |  |
| ﻿Δ 0-12 weeks | 0.01 ± 0.1 | 0.0 ± 0.2 | -0.01 (-0.1, 0.1) | 0.90 |
| Δ 12-24 weeks | 0.09 ± 0.2 | 0.03 ± 0.2 | -0.1 (-0.2, 0.05) | 0.27 |
| **400 metre walk time (s)** |  |  |  |  |
| Baseline | 273.1 ± 55.8 | 261.6 ± 34.7 |  |  |
| ﻿Δ 0-12 weeks | -7.4 ± 20.6 | -2.5 ± 16.3 | 4.9 (-5.2, 15.0) | 0.34 |
| Δ 12-24 weeks | 3.4 ± 26.5 | 1.3 ± 27.0 | -2.1 (-16.3, 12.1) | 0.77 |
| **SPPB score** |  |  |  |  |
| Baseline | 10.9 ± 1.5 | 11.4 ± 1.0 |  |  |
| ﻿Δ 0-12 weeks | 0.1 ± 1.5 | 0.1 ± 1.0 | -0.04 (-0.8, 0.7) | 0.91 |
| Δ 12-24 weeks | 0.3 ± 2.5 | -0.1 ± 1.5 | -0.4 (-1.3, 0.6) | 0.48 |

Baseline values are presented as mean ± SD. Net differences are means with 95% confidence intervals. Bold indicates statistical significance (P<0.05). HOMA-IR - homeostatic model assessment of insulin resistance; HDL - high-density lipoprotein; LDL - low-density lipoprotein; BMI – body mass index; ALM – appendicular lean mass; ALM/H^2^ – appendicular lean mass/height^2^; SPPB – short physical performance battery.

**Supplementary Table S2**. Mean baseline values and per-protocol analysis of between-group changes in blood biochemistry, body composition and physical function after 12 and 24 weeks in placebo and vitamin D groups.

|  | Placebo  N=10 | Vitamin D  N=13 | Net Difference  (95%CI) | Group x Time  (P-value) |
| --- | --- | --- | --- | --- |
| **Blood biochemistry** |  |  |  |  |
| **25-hydroxyvitamin D (nmol/L)** |  |  |  |  |
| Baseline | 46.2 ± 4.5 | 42.2 ± 8.4 |  |  |
| ﻿Δ 0-12 weeks | 4.8 ± 13.0 | 46.5 ± 15.7 | **41.7 (28.9, 54.5)** | **<0.001** |
| Δ 12-24 weeks | 6.2 ± 12.1 | 9.6 ± 11.0 | 3.4 (-6.6, 13.5) | 0.54 |
| **Fasting glucose (mmol/L)** |  |  |  |  |
| Baseline | 5.6 ± 1.0 | 5.3 ± 0.8 |  |  |
| ﻿Δ 0-12 weeks | 0.2 ± 0.5 | 0.1 ± 0.5 | -0.05 (-0.5, 0.4) | 0.78 |
| Δ 12-24 weeks | 0.03 ± 0.4 | 0.2 ± 0.5 N=11 | 0.1 (-0.3, 0.6) | 0.54 |
| **Fasting insulin (mUL)** |  |  |  |  |
| Baseline | 9.5 ± 6.8 | 7.3 ± 4.4 |  |  |
| ﻿Δ 0-12 weeks | 1.1 ± 3.5 | 1.2 ± 4.2 | 0.1 (-3.3, 3.5) | 0.58 |
| Δ 12-24 weeks | -2.2 ± 3.3 | -1.2 ± 3.1 | 1.0 (-1.7, 3.8) | 0.49 |
| **HOMA-IR (units)** |  |  |  |  |
| Baseline | 2.5 ± 2.4 | 1.7 ± 1.1 |  |  |
| ﻿Δ 0-12 weeks | 0.5 ± 1.2 | 0.4 ± 1.3 | -0.1 (-1.2, 1.0) | 0.61 |
| Δ 12-24 weeks | -0.6 ± 1.0 | -0.2 ± 1.0 N=11 | 0.4 (-0.5, 1.3) | 0.36 |
| **Triglycerides (mmol/L)** |  |  |  |  |
| Baseline | 1.2 ± 0.4 | 1.4 ± 0.5 |  |  |
| ﻿Δ 0-12 weeks | 0.04 ± 0.3 | -0.2 ± 0.5 | -0.2 (-0.5, 0.2) | 0.32 |
| Δ 12-24 weeks | -0.2 ± 0.3 | 0.1 ± 0.4 | 0.2 (-0.1, 0.5) | 0.15 |
| **HDL (mmol/L)** |  |  |  |  |
| Baseline | 1.6 ± 0.4 | 1.7 ± 0.3 |  |  |
| ﻿Δ 0-12 weeks | -0.01 ± 0.2 | -0.05 ± 0.1 | -0.04 (-0.2, 0.1) | 0.43 |
| Δ 12-24 weeks | 0.1 ± 0.3 | 0.0 ± 0.2 | -0.1 (-0.3, 0.1) | 0.21 |
| **LDL (mmol/L)** |  |  |  |  |
| Baseline | 4.0 ± 0.6 | 3.4 ± 0.9 |  |  |
| ﻿Δ 0-12 weeks | 0.1 ± 0.5 | 0.02 ± 0.4 | -0.03 (-0.4, 0.4) | 0.73 |
| Δ 12-24 weeks | 0.1 ± 0.5 | 0.2 ± 0.5 | 0.1 (-0.3, 0.5) | 0.45 |
| **Body composition** |  |  |  |  |
| **Weight (kg)** |  |  |  |  |
| Baseline | 79 ± 14.9 | 78.9 ± 16.6 |  |  |
| ﻿Δ 0-12 weeks | -0.5 ± 1.2 | -0.1 ± 1.5 | 0.5 (-0.8, 1.7) | 0.57 |
| Δ 12-24 weeks | 0.02 ± 1.1 | 0.2 ± 2.5 | 0.1 (-1.6, 1.9) | 0.87 |
| **BMI (kg/m^2^)** |  |  |  |  |
| Baseline | 28.1 ± 4.7 | 29.0 ± 4.6 |  |  |
| ﻿Δ 0-12 weeks | -0.2 ± 0.5 | 0.1 ± 0.7 | 0.3 (-0.3, 0.8) | 0.77 |
| Δ 12-24 weeks | 0.2 ± 0.4 | 0.1 ± 1.0 | -0.2 (-0.9, 0.5) | 0.59 |
| **Waist circumference (cm)** |  |  |  |  |
| Baseline | 99.3 ± 10.3 | 101.6 ± 16.3 |  |  |
| ﻿Δ 0-12 weeks | -1.7 ± 3.2 | -0.6 ± 4.4 | 1.1 (-2.3, 4.6) | 0.49 |
| Δ 12-24 weeks | -0.5 ± 4.2 | -3.6 ± 6.9 | -3.2 (-8.3, 2.0) | 0.14 |
| **Hip circumference (cm)** |  |  |  |  |
| Baseline | 106.2 ± 8.4 | 108.9 ± 12.5 |  |  |
| ﻿Δ 0-12 weeks | 0.1 ± 2.5 | 0.3 ± 3.0 | 0.2 (-2.3, 2.6) | 0.88 |
| Δ 12-24 weeks | -1.6 ± 3.2 | 0.7 ± 2.8 | 2.4 (-0.3, 5.0) | 0.08 |
| **Waist Hip Ratio** |  |  |  |  |
| Baseline | 0.93 ± 0.04 | 0.93 ± 0.1 |  |  |
| ﻿Δ 0-12 weeks | -0.02 ± 0.03 | -0.01 ± 0.04 | 0.01 (-0.02, 0.04) | 0.59 |
| Δ 12-24 weeks | 0.01 ± 0.03 | -0.03 ± 0.1 | **-0.04 (-0.1, -0.002)** | **0.04** |
| **ALM (kg)** |  |  |  |  |
| Baseline | 23.0 ± 7.0 | 19.3 ± 3.9 |  |  |
| ﻿Δ 0-12 weeks | 0.1 ± 0.6 | 0.2 ± 0.7 | 0.1 (-0.5, 0.7) | 0.82 |
| Δ 12-24 weeks | 0.1 ± 0.6 | -0.03 ± 0.4 | -0.1 (-0.5, 0.4) | 0.83 |
| **ALM/height^2^ (kg)** |  |  |  |  |
| Baseline | 8.0 ± 1.7 | 7.2 ± 0.8 |  |  |
| ﻿Δ 0-12 weeks | 0.02 ± 0.2 | 0.1 ± 0.2 | 0.05 (-0.2, 0.3) | 0.88 |
| Δ 12-24 weeks | 0.01 ± 0.2 | -0.01 ± 0.1 | -0.02 (-0.2, 0.1) | 0.91 |
| **Fat mass (kg)** |  |  |  |  |
| Baseline | 26.4 ± 7.0 | 31.4 ± 10.2 |  |  |
| ﻿Δ 0-12 weeks | -0.5 ± 1.8 | -0.2 ± 2.0 | 0.3 (-1.4, 2.1) | 0.83 |
| Δ 12-24 weeks | 0.3 ± 0.7 | 0.2 ± 2.3 | -0.1 (-1.7, 1.5) | 0.95 |
| **Body fat percentage** |  |  |  |  |
| Baseline | 35.6 ± 8.1 | 42.1 ± 6.4 |  |  |
| ﻿Δ 0-12 weeks | -0.6 ± 1.7 | -0.1 ± 1.9 | 0.5 (-1.2, 2.1) | 0.84 |
| Δ 12-24 weeks | 0.3 ± 1.2 | 0.0 ± 1.7 | -0.3 (-1.6, 1.1) | 0.71 |
| **Visceral adipose tissue (cm^2^)** |  |  |  |  |
| Baseline | 126.8 ± 54.5 | 125.4 ± 59.9 |  |  |
| ﻿Δ 0-12 weeks | -1.7 ± 11.4 | -5.0 ± 21.8 | -3.3 (-19.5, 12.8) | 0.40 |
| Δ 12-24 weeks | 7.0 ± 6.2 | 0.4 ± 15.7 | -6.6 (-17.6, 4.4) | 0.28 |
| **Calf muscle density (mg/cm^3^)** |  |  |  |  |
| Baseline | 75.1 ± 1.8 | 74.5 ± 2.2 |  |  |
| ﻿Δ 0-12 weeks | -0.2 ± 1.1 N=9 | -0.7 ± 2.0 N=12 | -0.5 (-2.3, 1.3) | 0.94 |
| Δ 12-24 weeks | -0.3 ± 1.1 N=8 | -0.1 ± 1.0 N=12 | 0.2 (-1.1, 1.5) | 0.83 |
| **Physical function** |  |  |  |  |
| **Hand grip strength (kg)** |  |  |  |  |
| Baseline | 39.6 ± 16.7 | 32.3 ± 8.6 |  |  |
| ﻿Δ 0-12 weeks | 1.8 ± 3.6 N=9 | -0.4 ± 3.1 | -2.2 (-5.2, 0.8) | 0.66 |
| Δ 12-24 weeks | -2.4 ± 2.5 N=9 | -0.1 ± 2.6 | 2.3 (-0.1, 4.6) | 0.17 |
| **Upper-limb relative strength** |  |  |  |  |
| Baseline | 12.9 ± 2.3 | 12.9 ± 2.2 |  |  |
| ﻿Δ 0-12 weeks | 0.4 ± 1.4 N=9 | -0.2 ± 1.2 | -0.6 (-1.8, 0.6) | 0.77 |
| Δ 12-24 weeks | -0.5 ± 0.8 N=9 | 0.3 ± 1.0 | 0.9 (-0.01, 1.7) | 0.27 |
| **Chair stand time (s)** |  |  |  |  |
| Baseline | 7.8 ± 3.1 | 9.3 ± 2.3 |  |  |
| ﻿Δ 0-12 weeks | -0.7 ± 1.9 | -1.5 ± 1.2 | -0.8 (-2.2, 0.5) | 0.74 |
| Δ 12-24 weeks | -1.2 ± 2.0 | -0.7 ± 1.6 | 0.5 (-1.0, 2.1) | 0.46 |
| **Stair climb time (s)** |  |  |  |  |
| Baseline | 3.6 ± 0.7 | 3.9 ± 0.5 |  |  |
| ﻿Δ 0-12 weeks | 0.04 ± 0.4 | -0.3 ± 0.6 | -0.4 (-0.8, 0.1) | 0.82 |
| Δ 12-24 weeks | -0.2 ± 0.4 | 0.1 ± 0.6 | 0.3 (-0.2, 0.8) | 0.20 |
| **Gait speed (m/s)** |  |  |  |  |
| Baseline | 0.9 ± 0.1 | 0.9 ± 0.2 |  |  |
| ﻿Δ 0-12 weeks | -0.03 ± 0.1 N=9 | -0.01 ± 0.2 N=11 | 0.02 (-0.1, 0.2) | 0.23 |
| Δ 12-24 weeks | 0.1 ± 0.1 N=9 | 0.02 ± 0.2 N=11 | -0.1 (-0.2, 0.04) | 0.12 |
| **400 metre walk time (s)** |  |  |  |  |
| Baseline | 243.6 ± 31.4 | 261.5 ± 30.0 |  |  |
| ﻿Δ 0-12 weeks | -10.7 ± 12.8 | -3.8 ± 18.0 | 6.9 (-7.1, 20.9) | 0.53 |
| Δ 12-24 weeks | -0.1 ± 10.6 | -0.9 ± 21.3 | -0.8 (-16.1, 14.6) | 0.92 |
| **SPPB score** |  |  |  |  |
| Baseline | 11.6 ± 1.0 | 11.6 ± 0.7 |  |  |
| ﻿Δ 0-12 weeks | 0.0 ± 0.5 N=9 | -0.1 ± 1.3 | -0.1 (-1.0, 0.8) | 0.13 |
| Δ 12-24 weeks | 0.3 ± 1.0 N=9 | 0.4 ± 1.2 | 0.1 (-1.0, 1.1) | 0.26 |

Baseline values are mean ± SD. Net differences are means with 95% confidence intervals. Bold indicates statistical significance (P<0.05). HOMA-IR - homeostatic model assessment of insulin resistance; HDL - high-density lipoprotein; LDL - low-density lipoprotein; BMI – body mass index; ALM – appendicular lean mass; ALM/H^2^ – appendicular lean mass/ height^2^; SPPB – short physical performance battery

**Supplementary Table S3**. Mean baseline values and within- and between-group changes in blood biochemistry, body composition and physical function after 12 and 24 weeks in women randomized to placebo or vitamin D.

|  | Placebo  N=15 | Vitamin D  N=16 | Net Difference  (95%CI) | Group x Time  (P-value) |
| --- | --- | --- | --- | --- |
| **Blood biochemistry** |  |  |  |  |
| **25-hydroxyvitamin D (nmol/L)** |  |  |  |  |
| Baseline | 42.7 ± 9.7 | 38.7 ± 8.1 |  |  |
| ﻿Δ 0-12 weeks | -0.8 ± 9.3 | 46.3 ± 15.4 | **47.1 (36.7, 57.5)** | **<0.001** |
| Δ 12-24 weeks | 3.7 ± 7.9 | 9.4 ± 11.1 | 5.7 (-3.2, 14.6) | 0.20 |
| **Fasting glucose (mmol/L)** |  |  |  |  |
| Baseline | 5.4 ± 0.7 | 5.3 ± 0.9 |  |  |
| ﻿Δ 0-12 weeks | -0.1 ± 0.4 | 0.2 ± 0.5 | 0.2 (-0.2, 0.6) | 0.23 |
| Δ 12-24 weeks | -0.1 ± 0.5 N=7 | 0.03 ± 0.5 N=12 | 0.1 (-0.3, 0.6) | 0.55 |
| **Fasting insulin (mUL)** |  |  |  |  |
| Baseline | 10.5 ± 7.1 | 8.8 ± 5.1 |  |  |
| ﻿Δ 0-12 weeks | 0.9 ± 3.9 | -0.1 ± 3.7 | -1.0 (-4.1, 2.0) | 0.49 |
| Δ 12-24 weeks | -1.8 ± 3.1 | -0.5 ± 3.4 | 1.3 (-1.6, 4.2) | 0.36 |
| **HOMA-IR (units)** |  |  |  |  |
| Baseline | 2.6 ± 1.9 | 2.1 ± 1.4 |  |  |
| ﻿Δ 0-12 weeks | 0.3 ± 1.1 | 0.1 ± 1.0 | -0.2 (-1.0, 0.6) | 0.61 |
| Δ 12-24 weeks | -0.6 ± 1.1 N=7 | 0.1 ± 1.1 N=12 | 0.7 (-0.4, 1.8) | 0.22 |
| **Triglycerides (mmol/L)** |  |  |  |  |
| Baseline | 1.4 ± 1.0 | 1.4 ± 0.5 |  |  |
| ﻿Δ 0-12 weeks | 0.1 ± 0.5 | -0.1 ± 0.4 | -0.2 (-0.6, 0.1) | 0.20 |
| Δ 12-24 weeks | -0.2 ± 0.5 | 0.1 ± 0.4 | 0.3 (-0.1, 0.7) | 0.13 |
| **HDL (mmol/L)** |  |  |  |  |
| Baseline | 1.6 ± 0.3 | 1.7 ± 0.3 |  |  |
| ﻿Δ 0-12 weeks | -0.1 ± 0.2 | -0.1 ± 0.1 | 0.02 (-0.1, 0.1) | 0.79 |
| Δ 12-24 weeks | 0.1 ± 0.3 | 0.01 ± 0.2 | -0.1 (-0.3, 0.1) | 0.45 |
| **LDL (mmol/L)** |  |  |  |  |
| Baseline | 3.6 ± 0.7 | 3.6 ± 0.9 |  |  |
| ﻿Δ 0-12 weeks | -0.1 ± 0.3 | -0.1 ± 0.5 | 0.0 (-0.3, 0.3) | 0.96 |
| Δ 12-24 weeks | 0.04 ± 0.4 | 0.2 ± 0.5 | 0.1 (-0.3, 0.6) | 0.48 |
| **Body composition** |  |  |  |  |
| **Weight (kg)** |  |  |  |  |
| Baseline | 85.1 ± 18.4 | 81.5 ± 18.9 |  |  |
| ﻿Δ 0-12 weeks | 0.5 ± 4.1 | -0.2 ± 1.6 | -0.8 (-3.2, 1.7) | 0.52 |
| Δ 12-24 weeks | -0.02 ± 1.3 | -0.2 ± 2.3 | -0.1 (-1.9, 1.6) | 0.86 |
| **BMI (kg/m^2^)** |  |  |  |  |
| Baseline | 33.2 ± 7.4 | 30.7 ± 5.5 |  |  |
| ﻿Δ 0-12 weeks | 0.2 ± 1.7 | -0.1 ± 0.8 | -0.3 (-1.3, 0.7) | 0.55 |
| Δ 12-24 weeks | 0.1 ± 0.6 | -0.02 ± 1.0 | -0.1 (-0.9, 0.6) | 0.76 |
| **Waist circumference (cm)** |  |  |  |  |
| Baseline | 105.9 ± 13.8 | 103.3 ± 14.6 |  |  |
| ﻿Δ 0-12 weeks | -2.2 ± 6.0 | -0.8 ± 4.3 | 1.4 (-2.7, 5.5) | 0.49 |
| Δ 12-24 weeks | 2.2 ± 7.5 | -4.5 ± 6.2 | **-6.8 (-12.8, -0.8)** | **0.03** |
| **Hip circumference (cm)** |  |  |  |  |
| Baseline | 116.5 ± 12 | 112.9 ± 14.3 |  |  |
| ﻿Δ 0-12 weeks | 0.1 ± 3.3 | -0.4 ± 3.3 | -0.5 (-3.1, 2.1) | 0.70 |
| Δ 12-24 weeks | -0.7 ± 3.2 | 0.2 ± 2.5 | 0.9 (-1.5, 3.4) | 0.42 |
| **Waist Hip Ratio** |  |  |  |  |
| Baseline | 0.9 ± 0.1 | 0.9 ± 0.1 |  |  |
| ﻿Δ 0-12 weeks | -0.02 ± 0.1 | -0.01 ± 0.04 | 0.01 (-0.02, 0.1) | 0.48 |
| Δ 12-24 weeks | 0.02 ± 0.1 | -0.04 ± 0.1 | **-0.1 (-0.1, -0.01)** | **0.02** |
| **ALM (kg)** |  |  |  |  |
| Baseline | 19.5 ± 3.1 | 19.1 ± 4.1 |  |  |
| ﻿Δ 0-12 weeks | 0.3 ± 1.7 | 0.1 ± 0.6 | -0.2 (-1.1, 0.7) | 0.67 |
| Δ 12-24 weeks | -0.02 ± 0.4 | -0.05 ± 0.3 | -0.03 (-0.3, 0.3) | 0.86 |
| **ALM/height^2^ (kg)** |  |  |  |  |
| Baseline | 7.6 ± 1.1 | 7.2 ± 1.0 |  |  |
| ﻿Δ 0-12 weeks | 0.1 ± 0.6 | 0.04 ± 0.2 | -0.1 (-0.4, 0.3) | 0.68 |
| Δ 12-24 weeks | -0.01 ± 0.1 | -0.01 ± 0.1 | 0.0 (-0.1, 0.1) | 0.94 |
| **Fat mass (kg)** |  |  |  |  |
| Baseline | 38.4 ± 11.8 | 35.3 ± 11.3 |  |  |
| ﻿Δ 0-12 weeks | -0.2 ± 2.3 | -0.3 ± 1.8 | -0.1 (-1.8, 1.6) | 0.91 |
| Δ 12-24 weeks | 0.3 ± 1.5 | -0.1 ± 2.0 | -0.4 (-2.1, 1.4) | 0.65 |
| **Body fat percentage** |  |  |  |  |
| Baseline | 46.3 ± 4.8 | 44.9 ± 5.8 |  |  |
| ﻿Δ 0-12 weeks | -0.5 ± 1.3 | -0.2 ± 1.7 | 0.3 (-1.0, 1.5) | 0.66 |
| Δ 12-24 weeks | 0.6 ± 1.1 | -0.2 ± 1.5 | -0.8 (-2.1, 0.5) | 0.22 |
| **Visceral adipose tissue (cm^2^)** |  |  |  |  |
| Baseline | 136.5 ± 56.3 | 132.8 ± 57.3 |  |  |
| ﻿Δ 0-12 weeks | -5.0 ± 15.0 | -4.6 ± 19.9 | 0.4 (-14.5, 15.4) | 0.95 |
| Δ 12-24 weeks | 1.3 ± 14.9 | 0.4 ± 15.6 | -0.9 (-15.4, 13.5) | 0.89 |
| **Calf muscle density (mg/cm^3^)** |  |  |  |  |
| Baseline | 72.7 ± 2.3 | 73.5 ± 2.8 |  |  |
| ﻿Δ 0-12 weeks | -0.4 ± 1.2 N=10 | -0.7 ± 1.6 N=14 | -0.4 (-1.9, 1.1) | 0.60 |
| Δ 12-24 weeks | 0.3 ± 1.2 N=6 | -0.1 ± 0.9 N=13 | -0.4 (-1.6, 0.8) | 0.48 |
| **Physical function** |  |  |  |  |
| **Hand grip strength (kg)** |  |  |  |  |
| Baseline | 24 ± 8.2 | 26.9 ± 6.7 |  |  |
| ﻿Δ 0-12 weeks | 0.8 ± 3.0 | -0.1 ± 2.8 | -0.9 (-3.4, 1.6) | 0.45 |
| Δ 12-24 weeks | -3.0 ± 3.0 N=7 | -0.3 ± 2.1 | **2.7 (0.3, 5.0)** | **0.03** |
| **Upper-limb relative strength** |  |  |  |  |
| Baseline | 10.4 ± 3.6 | 11.7 ± 3.2 |  |  |
| ﻿Δ 0-12 weeks | 0.4 ± 1.7 | 0.0 ± 1.4 | -0.4 (-1.8, 1.0) | 0.54 |
| Δ 12-24 weeks | -1.2 ± 1.5 N=7 | 0.1 ± 1.1 | **1.3 (0.1, 2.5)** | **0.04** |
| **Chair stand time (s)** |  |  |  |  |
| Baseline | 10.4 ± 3.3 | 9.6 ± 2.2 |  |  |
| ﻿Δ 0-12 weeks | -1.0 ± 2.4 | -1.4 ± 1.4 | -0.4 (-1.9, 1.2) | 0.63 |
| Δ 12-24 weeks | -1.7 ± 1.8 | -0.2 ± 1.8 N=13 | 1.5 (-0.2, 3.1) | 0.07 |
| **Stair climb time (s)** |  |  |  |  |
| Baseline | 4.8 ± 1.4 | 4.4 ± 1.3 |  |  |
| ﻿Δ 0-12 weeks | 0.3 ± 0.9 | -0.1 ± 0.6 | -0.4 (-1.0, 0.2) | 0.15 |
| Δ 12-24 weeks | -0.05 ± 1.1 | 0.3 ± 1.0 | 0.3 (-0.6, 1.3) | 0.46 |
| **Gait speed (m/s)** |  |  |  |  |
| Baseline | 0.83 ± 0.2 | 0.92 ± 0.2 |  |  |
| ﻿Δ 0-12 weeks | 0.02 ± 0.1 | -0.02 ± 0.1 N=13 | -0.03 (-0.1, 0.1) | 0.55 |
| Δ 12-24 weeks | 0.1 ± 0.2 | -0.03 ± 0.2 N=12 | -0.1 (-0.3, 0.1) | 0.17 |
| **400 metre walk time (s)** |  |  |  |  |
| Baseline | 294.4 ± 59.8 | 269.8 ± 39.1 |  |  |
| ﻿Δ 0-12 weeks | -6.5 ± 23.6 | 0.3 ± 17.1 | 6.8 (-9.7, 23.2) | 0.40 |
| Δ 12-24 weeks | 0.9 ± 9.7 N=8 | 5.1 ± 28 | 4.3 (-17.2, 25.8) | 0.68 |
| **SPPB score** |  |  |  |  |
| Baseline | 10.7 ± 1.7 | 11.5 ± 1.0 |  |  |
| ﻿Δ 0-12 weeks | 0.5 ± 1.2 | -0.2 ± 1.1 | -0.7 (-1.6, 0.3) | 0.17 |
| Δ 12-24 weeks | 0.8 ± 1.2 N=8 | -0.1 ± 1.6 | -0.8 (-2.2, 0.6) | 0.23 |

Baseline values are mean ± SD. Net differences are means with 95% confidence intervals. Bold indicates statistical significance (P<0.05). HOMA-IR – homeostatic model assessment of insulin resistance; HDL – high-density lipoprotein; LDL – low-density lipoprotein; BMI – body mass index; ALM – appendicular lean mass; ALM/H^2^ – appendicular lean mass/ height^2^; SPPB – short physical performance battery. Placebo: ﻿Δ 0-12 weeks N=12, Δ 12-24 weeks N=9; Vitamin D: Δ 0-12 weeks N=15, Δ 12-24 weeks N=14.

**Supplementary Table S4**. Mean baseline values and within- and between-group changes in blood biochemistry, body composition and physical function after 12 and 24 weeks in men randomized to placebo or vitamin D.

|  | Placebo  N=9 | Vitamin D  N=10 | Net Difference  (95%CI) | Group x Time  (P-value) |
| --- | --- | --- | --- | --- |
| **Blood biochemistry** |  |  |  |  |
| **25-hydroxyvitamin D (nmol/L)** |  |  |  |  |
| Baseline | 40.3 ± 9.9 | 40.7 ± 14.4 |  |  |
| ﻿Δ 0-12 weeks | 7.5 ± 12.0 | 39.9 ± 21.7 | **32.4 (14.2, 50.6)** | **<0.001** |
| Δ 12-24 weeks | 8.3 ± 15.1 | 4.3 ± 5.9 | -4.0 (-15.7, 7.8) | 0.48 |
| **Fasting glucose (mmol/L)** |  |  |  |  |
| Baseline | 5.5 ± 1.1 | 6.7 ± 1.4 |  |  |
| ﻿Δ 0-12 weeks | 0.1 ± 0.5 | 0.4 ± 2.2 | 0.3 (-1.4, 1.9) | 0.76 |
| Δ 12-24 weeks | 0.2 ± 0.3 | 0.9 ± 1.0 | 0.7 (-0.2, 1.5) | 0.11 |
| **Fasting insulin (mUL)** |  |  |  |  |
| Baseline | 7.9 ± 6.4 | 9.8 ± 6.2 |  |  |
| ﻿Δ 0-12 weeks | 0.1 ± 2.6 | 1.9 ± 4.3 | 1.8 (-1.9, 5.5) | 0.31 |
| Δ 12-24 weeks | -1.6 ± 2.4 | 1.1 ± 3.9 | 2.7 (-0.9, 6.4) | 0.12 |
| **HOMA-IR (units)** |  |  |  |  |
| Baseline | 2.2 ± 2.5 | 3.0 ± 2.2 |  |  |
| ﻿Δ 0-12 weeks | 0.2 ± 1.1 | 1.5 ± 3.5 | 1.3 (-1.4, 4.0) | 0.33 |
| Δ 12-24 weeks | -0.4 ± 0.7 | 1.4 ± 2.8 | 1.8 (-0.5, 4.1) | 0.12 |
| **Triglycerides (mmol/L)** |  |  |  |  |
| Baseline | 1.4 ± 0.4 | 1.5 ± 0.5 |  |  |
| ﻿Δ 0-12 weeks | -0.1 ± 0.3 | 0.2 ± 0.6 | 0.30 (-0.2, 0.7) | 0.28 |
| Δ 12-24 weeks | -0.2 ± 0.2 | -0.1 ± 0.2 | 0.03 (-0.2, 0.2) | 0.79 |
| **HDL (mmol/L)** |  |  |  |  |
| Baseline | 1.3 ± 0.1 | 1.2 ± 0.2 |  |  |
| ﻿Δ 0-12 weeks | -0.01 ± 0.3 | -0.1 ± 0.1 | -0.1 (-0.3, 0.2) | 0.63 |
| Δ 12-24 weeks | 0.1 ± 0.4 | 0.1 ± 0.1 | -0.04 (-0.3, 0.2) | 0.78 |
| **LDL (mmol/L)** |  |  |  |  |
| Baseline | 4.1 ± 0.6 | 2.9 ± 1.4 |  |  |
| ﻿Δ 0-12 weeks | 0.1 ± 0.6 | 0.04 ± 0.6 | -0.1 (-0.7, 0.5) | 0.74 |
| Δ 12-24 weeks | -0.1 ± 0.4 | 0.02 ± 0.6 | 0.1 (-0.5, 0.7) | 0.73 |
| **Body composition** |  |  |  |  |
| **Weight (kg)** |  |  |  |  |
| Baseline | 88.4 ± 16.4 | 87 ± 11.1 |  |  |
| ﻿Δ 0-12 weeks | -0.8 ± 2.2 | -0.4 ± 2.0 | 0.4 (-1.8, 2.5) | 0.72 |
| Δ 12-24 weeks | 0.01 ± 1.1 | -0.2 ± 1.8 | -0.2 (-1.8, 1.5) | 0.81 |
| **BMI (kg/m^2^)** |  |  |  |  |
| Baseline | 28.6 ± 3.8 | 28.5 ± 2.5 |  |  |
| ﻿Δ 0-12 weeks | -0.2 ± 0.7 | -0.2 ± 0.8 | 0.03 (-0.7, 0.8) | 0.94 |
| Δ 12-24 weeks | 0.2 ± 0.3 | -0.07 ± 0.6 | -0.2 (-0.8, 0.3) | 0.39 |
| **Waist circumference (cm)** |  |  |  |  |
| Baseline | 100.7 ± 9.3 | 101.9 ± 8.5 |  |  |
| ﻿Δ 0-12 weeks | -0.7 ± 2.5 | -0.7 ± 3.0 | -0.03 (-2.8, 2.8) | 0.99 |
| Δ 12-24 weeks | 0.5 ± 1.9 | -1.0 ± 4.4 | -1.5 (-5.3, 2.4) | 0.43 |
| **Hip circumference (cm)** |  |  |  |  |
| Baseline | 107.1 ± 8.8 | 105.3 ± 4.5 |  |  |
| ﻿Δ 0-12 weeks | 0.04 ± 1.8 | -0.3 ± 2.7 | -0.3 (-2.7, 2.1) | 0.79 |
| Δ 12-24 weeks | -1.2 ± 2.6 | 1.7 ± 3.3 | 2.9 (-0.4, 6.1) | 0.08 |
| **Waist Hip Ratio** |  |  |  |  |
| Baseline | 0.9 ± 0.05 | 1.0 ± 0.1 |  |  |
| ﻿Δ 0-12 weeks | -0.01 ± 0.03 | -0.004 ± 0.02 | 0.003 (-0.02, 0.03) | 0.80 |
| Δ 12-24 weeks | 0.01 ± 0.02 | -0.02 ± 0.04 | **-0.04 (-0.1, -0.003)** | **0.04** |
| **ALM (kg)** |  |  |  |  |
| Baseline | 28.1 ± 5.2 | 27.5 ± 3.5 |  |  |
| ﻿Δ 0-12 weeks | 0.001 ± 0.5 | -0.1 ± 0.7 | -0.1 (-0.8, 0.5) | 0.69 |
| Δ 12-24 weeks | 0.2 ± 0.8 | 0.003 ± 0.4 | -0.2 (-0.9, 0.4) | 0.44 |
| **ALM/height^2^ (kg)** |  |  |  |  |
| Baseline | 9.1 ± 1.3 | 9.0 ± 0.9 |  |  |
| ﻿Δ 0-12 weeks | 0.005 ± 0.2 | -0.04 ± 0.2 | -0.04 (-0.2, 0.2) | 0.69 |
| Δ 12-24 weeks | 0.1 ± 0.3 | -0.001 ± 0.1 | -0.1 (-0.3, 0.1) | 0.48 |
| **Fat mass (kg)** |  |  |  |  |
| Baseline | 25.8 ± 7.0 | 25.4 ± 5.1 |  |  |
| ﻿Δ 0-12 weeks | -0.7 ± 1.8 | -0.5 ± 1.6 | 0.2 (-1.5, 2.0) | 0.79 |
| Δ 12-24 weeks | 0.1 ± 0.6 | -0.04 ± 1.8 | -0.1 (-1.6, 1.4) | 0.86 |
| **Body fat percentage** |  |  |  |  |
| Baseline | 30.5 ± 3.7 | 31.1 ± 3.6 |  |  |
| ﻿Δ 0-12 weeks | -0.6 ± 1.5 | -0.5 ± 1.6 | 0.1 (-1.5, 1.6) | 0.94 |
| Δ 12-24 weeks | -0.02 ± 1.2 | 0.1 ± 1.4 | 0.1 (-1.3, 1.5) | 0.91 |
| **Visceral adipose tissue (cm^2^)** |  |  |  |  |
| Baseline | 161.1 ± 60.4 | 152.2 ± 39.5 |  |  |
| ﻿Δ 0-12 weeks | -2.9 ± 16.9 | -2.7 ± 14.3 | 0.2 (-15.9, 16.3) | 0.98 |
| Δ 12-24 weeks | 8.5 ± 7.4 | -2.3 ± 22.3 | -10.9 (-29.8, 8.1) | 0.24 |
| **Calf muscle density (mg/cm^3^)** |  |  |  |  |
| Baseline | 76.1 ± 1.0 | 75.5 ± 1.6 |  |  |
| ﻿Δ 0-12 weeks | -0.6 ± 0.8 N=7 | -0.6 ± 2.2 | 0.03 (-2.5, 2.6) | 0.98 |
| Δ 12-24 weeks | -1.5 ± 0.3 N=6 | 0.4 ± 1.1 N=6 | **1.8 (0.3, 3.4)** | **0.03** |
| **Physical function** |  |  |  |  |
| **Hand grip strength (kg)** |  |  |  |  |
| Baseline | 45.3 ± 13.4 | 44.1 ± 10.9 |  |  |
| ﻿Δ 0-12 weeks | 1.9 ± 4.0 | 1.8 ± 4.2 | -0.1 (-4.2, 4.1) | 0.96 |
| Δ 12-24 weeks | -1.8 ± 3.4 | 0.3 ± 3.8 | 2.1 (-1.8, 6.1) | 0.26 |
| **Upper-limb relative strength** |  |  |  |  |
| Baseline | 11.9 ± 3.0 | 11.5 ± 2.3 |  |  |
| ﻿Δ 0-12 weeks | 0.4 ± 1.4 | 0.6 ± 1.2 | 0.2 (-1.1, 1.5) | 0.73 |
| Δ 12-24 weeks | -0.4 ± 0.9 | 0.2 ± 0.9 | 0.6 (-0.4, 1.6) | 0.20 |
| **Chair stand time (s)** |  |  |  |  |
| Baseline | 10.1 ± 8.0 | 8.5 ± 2.7 |  |  |
| ﻿Δ 0-12 weeks | -1.2 ± 2.3 | -1.1 ± 2.8 | 0.03 (-2.7, 2.7) | 0.98 |
| Δ 12-24 weeks | -0.8 ± 1.3 | -0.02 ± 1.6 | 0.8 (-0.8, 2.4) | 0.31 |
| **Stair climb time (s)** |  |  |  |  |
| Baseline | 3.6 ± 0.7 | 3.9 ± 1.5 |  |  |
| ﻿Δ 0-12 weeks | 0.02 ± 0.4 | -0.4 ± 1.0 | -0.4 (-1.2, 0.4) | 0.30 |
| Δ 12-24 weeks | -0.01 ± 0.3 | 0.1 ± 0.5 | 0.1 (-0.3, 0.6) | 0.53 |
| **Gait speed (m/s)** |  |  |  |  |
| Baseline | 0.89 ± 0.2 | 0.85 ± 0.2 |  |  |
| ﻿Δ 0-12 weeks | -0.05 ± 0.1 N=7 | 0.01 ± 0.2 N=9 | 0.1 (-0.1, 0.2) | 0.50 |
| Δ 12-24 weeks | 0.1 ± 0.1 N=6 | 0.08 ± 0.2 N=8 | 0.02 (-0.1, 0.2) | 0.70 |
| **400 metre walk time (s)** |  |  |  |  |
| Baseline | 239.9 ± 29.4 | 248.5 ± 22.1 |  |  |
| ﻿Δ 0-12 weeks | -10.0 ± 9.7 | -6.4 ± 11.6 | 3.6 (-7.3, 14.5) | 0.49 |
| Δ 12-24 weeks | 3.7 ± 15.1 | -3.1 ± 19.4 N=8 | -6.8 (-26.5, 12.8) | 0.47 |
| **SPPB score** |  |  |  |  |
| Baseline | 11.1 ± 1.7 | 11.3 ± 0.9 |  |  |
| ﻿Δ 0-12 weeks | 0.3 ± 0.5 | 0.4 ± 0.8 | 0.2 (-0.6, 0.9) | 0.66 |
| Δ 12-24 weeks | 0 ± 0 | 0.3 ± 0.5 | 0.3 (-0.1, 0.7) | 0.10 |

Baseline values are mean ± SD. Net differences are means with 95% confidence intervals. Bold indicates statistical significance (P<0.05). HOMA-IR - homeostatic model assessment of insulin resistance; HDL - high-density lipoprotein; LDL - low-density lipoprotein; BMI – body mass index; ALM – appendicular lean mass; ALM/H^2^ – appendicular lean mass/ height^2^; SPPB – short physical performance battery. Placebo: ﻿Δ 0-12 weeks N=8, Δ 12-24 weeks N=7; Vitamin D: Δ 12-24 weeks N=9.

**Supplementary Table S5**. Mean baseline values and within- and between-group changes in blood biochemistry, body composition and physical function after 12 and 24 weeks in overweight participants randomized to placebo or vitamin D.

|  | Placebo  N= 12 | Vitamin D  N=15 | Net Difference  (95%CI) | Group x Time  (P-value) |
| --- | --- | --- | --- | --- |
| **Blood biochemistry** |  |  |  |  |
| **25-hydroxyvitamin D (nmol/L)** |  |  |  |  |
| Baseline | 43.3 ± 7.5 | 36.9 ± 8.9 |  |  |
| ﻿Δ 0-12 weeks | 5.3 ± 11.4 | 47.1 ± 20.4 | **41.8 (27.7, 55.9)** | **<0.001** |
| Δ 12-24 weeks | 3.7 ± 11.3 | 9.4 ± 10.5 | 5.7 (-4.1, 15.5) | 0.24 |
| **Fasting glucose (mmol/L)** |  |  |  |  |
| Baseline | 5.2 ± 0.5 | 5.7 ± 1.2 |  |  |
| ﻿Δ 0-12 weeks | 0.1 ± 0.4 | 0.01 ± 1.0 | -0.1 (-0.7, 0.6) | 0.85 |
| Δ 12-24 weeks | 0.03 ± 0.3 N=8 | 0.2 ± 0.6 N=11 | 0.1 (-0.4, 0.6) | 0.56 |
| **Fasting insulin (mUL)** |  |  |  |  |
| Baseline | 6.2 ± 2.2 | 6.7 ± 4.4 |  |  |
| ﻿Δ 0-12 weeks | -0.5 ± 1.8 | 0.4 ± 2.9 | 0.9 (-1.1, 3.0) | 0.36 |
| Δ 12-24 weeks | -0.9 ± 1.2 | 0.4 ± 2.7 | 1.3 (-0.7, 3.4) | 0.19 |
| **HOMA-IR (units)** |  |  |  |  |
| Baseline | 1.5 ± 0.6 | 1.7 ± 1.4 |  |  |
| ﻿Δ 0-12 weeks | -0.1 ± 0.5 | 0.2 ± 1.2 | 0.3 (-0.5, 1.1) | 0.42 |
| Δ 12-24 weeks | -0.2 ± 0.3 N=8 | 0.5 ± 1.4 N=11 | 0.7 (-0.4, 1.8) | 0.20 |
| **Triglycerides (mmol/L)** |  |  |  |  |
| Baseline | 1.1 ± 0.3 | 1.3 ± 0.4 |  |  |
| ﻿Δ 0-12 weeks | 0.03 ± 0.3 | -0.1 ± 0.4 | -0.1 (-0.4, 0.2) | 0.33 |
| Δ 12-24 weeks | -0.2 ± 0.3 | -0.02 ± 0.4 | 0.2 (-0.1, 0.5) | 0.18 |
| **HDL (mmol/L)** |  |  |  |  |
| Baseline | 1.6 ± 0.4 | 1.5 ± 0.4 |  |  |
| ﻿Δ 0-12 weeks | -0.1 ± 0.2 | -0.1 ± 0.1 | -0.01 (-0.1, 0.1) | 0.89 |
| Δ 12-24 weeks | 0.1 ± 0.3 | 0.05 ± 0.1 | -0.1 (-0.3, 0.1) | 0.48 |
| **LDL (mmol/L)** |  |  |  |  |
| Baseline | 4.1 ± 0.5 | 3.3 ± 0.8 |  |  |
| ﻿Δ 0-12 weeks | 0.1 ± 0.5 | 0.1 ± 0.5 | 0.001 (-0.4, 0.4) | 0.99 |
| Δ 12-24 weeks | -0.1 ± 0.5 | 0.1 ± 0.6 | 0.1 (-0.4, 0.6) | 0.59 |
| **Body composition** |  |  |  |  |
| **Weight (kg)** |  |  |  |  |
| Baseline | 73.9 ± 9.6 | 73.4 ± 10.1 |  |  |
| ﻿Δ 0-12 weeks | 0.02 ± 2.0 | 0.1 ± 1.7 | 0.1 (-1.5, 1.6) | 0.93 |
| Δ 12-24 weeks | 0.2 ± 1.2 | -0.5 ± 1.6 | -0.7 (-2.0, 0.7) | 0.32 |
| **BMI (kg/m^2^)** |  |  |  |  |
| Baseline | 26.3 ± 1.8 | 26.6 ± 1.8 |  |  |
| ﻿Δ 0-12 weeks | 0.1 ± 0.9 | 0.05 ± 0.7 | -0.03 (-0.7, 0.6) | 0.93 |
| Δ 12-24 weeks | 0.2 ± 0.4 | -0.2 ± 0.6 | -0.5 (-1.0, 0.003) | 0.05 |
| **Waist circumference (cm)** |  |  |  |  |
| Baseline | 95.6 ± 4.8 | 94.7 ± 7.9 |  |  |
| ﻿Δ 0-12 weeks | -2.0 ± 4.0 | 0.2 ± 4.5 | 2.2 (-1.3, 5.7) | 0.21 |
| Δ 12-24 weeks | 0.1 ± 5.5 | -1.6 ± 4.9 | -1.7 (-6.3, 3.0) | 0.47 |
| **Hip circumference (cm)** |  |  |  |  |
| Baseline | 104.1 ± 6.0 | 103.6 ± 6.5 |  |  |
| ﻿Δ 0-12 weeks | -0.1 ± 2.1 | 0.2 ± 3.0 | 0.3 (-1.9, 2.5) | 0.80 |
| Δ 12-24 weeks | -1.3 ± 3.1 | 0.2 ± 3.0 | 1.5 (-1.2, 4.3) | 0.26 |
| **Waist Hip Ratio** |  |  |  |  |
| Baseline | 0.9 ± 0.04 | 0.9 ± 0.1 |  |  |
| ﻿Δ 0-12 weeks | -0.02 ± 0.04 | -0.001 ± 0.04 | 0.02 (-0.01, 0.1) | 0.25 |
| Δ 12-24 weeks | 0.01 ± 0.04 | -0.02 ± 0.04 | -0.03 (-0.1, 0.01) | 0.15 |
| **ALM (kg)** |  |  |  |  |
| Baseline | 21.4 ± 4.9 | 20.4 ± 4.7 |  |  |
| ﻿Δ 0-12 weeks | 0.2 ± 0.4 | 0.02 ± 0.6 | -0.1 (-0.6, 0.3) | 0.51 |
| Δ 12-24 weeks | -0.01 ± 0.7 | -0.1 ± 0.3 | -0.1 (-0.5, 0.4) | 0.82 |
| **ALM/height^2^ (kg)** |  |  |  |  |
| Baseline | 7.5 ± 1.2 | 7.3 ± 1.1 |  |  |
| ﻿Δ 0-12 weeks | 0.1 ± 0.2 | 0.01 ± 0.2 | -0.04 (-0.2, 0.1) | 0.58 |
| Δ 12-24 weeks | -0.01 ± 0.2 | -0.02 ± 0.1 | -0.01 (-0.2, 0.1) | 0.88 |
| **Fat mass (kg)** |  |  |  |  |
| Baseline | 24.8 ± 5.4 | 25.7 ± 5.3 |  |  |
| ﻿Δ 0-12 weeks | -0.9 ± 1.4 | -0.2 ± 1.4 | 0.7 (-0.4, 1.9) | 0.22 |
| Δ 12-24 weeks | 0.5 ± 1.0 | -0.5 ± 1.0 | **-1.0 (-2.0, -0.04)** | **0.04** |
| **Body fat percentage** |  |  |  |  |
| Baseline | 35.5 ± 7.2 | 37.4 ± 7.1 |  |  |
| ﻿Δ 0-12 weeks | -0.8 ± 1.4 | -0.2 ± 1.6 | 0.6 (-0.7, 1.8) | 0.34 |
| Δ 12-24 weeks | 0.4 ± 1.3 | -0.5 ± 1.1 | -0.9 (-2.0, 0.2) | 0.09 |
| **Visceral adipose tissue (cm^2^)** |  |  |  |  |
| Baseline | 106.7 ± 28.2 | 120.5 ± 49.2 |  |  |
| ﻿Δ 0-12 weeks | -4.6 ± 7.1 | 1.2 ± 14 | 5.8 (-3.7, 15.4) | 0.22 |
| Δ 12-24 weeks | 7.4 ± 5.3 | 1.5 ± 14.8 | -5.8 (-16.7, 5.1) | 0.28 |
| **Calf muscle density (mg/cm^3^)** |  |  |  |  |
| Baseline | 74.1 ± 2.1 | 75.3 ± 2.1 |  |  |
| ﻿Δ 0-12 weeks | -0.3 ± 1.3 N=9 | -0.8 ± 2.3 | -0.5 (-2.9, 1.8) | 0.63 |
| Δ 12-24 weeks | -0.4 ± 0.9 N=7 | 0.1 ± 1.0 N=11 | 0.5 (-0.7, 1.8) | 0.39 |
| **Physical function** |  |  |  |  |
| **Hand grip strength (kg)** |  |  |  |  |
| Baseline | 34.1 ± 14.1 | 31.7 ± 10.5 |  |  |
| ﻿Δ 0-12 weeks | 1.7 ± 2.9 N=10 | 0.8 ± 3.8 N=14 | -0.9 (-3.9, 2.0) | 0.52 |
| Δ 12-24 weeks | -2.9 ± 2.1 N=8 | 0.4 ± 3.3 | **3.3 (0.5, 6.1)** | **0.02** |
| **Upper-limb relative strength** |  |  |  |  |
| Baseline | 12.2 ± 3.7 | 11.9 ± 3.3 |  |  |
| ﻿Δ 0-12 weeks | 0.5 ± 1.4 N=10 | 0.4 ± 1.6 N=14 | -0.1 (-1.4, 1.2) | 0.85 |
| Δ 12-24 weeks | -0.7 ± 0.8 N=8 | 0.4 ± 1.2 | **1.1 (0.1, 2.1)** | **0.04** |
| **Chair stand time (s)** |  |  |  |  |
| Baseline | 9.7 ± 7.3 | 8.8 ± 2.6 |  |  |
| ﻿Δ 0-12 weeks | -0.7 ± 1.9 | -1.3 ± 2.4 | -0.6 (-2.5, 1.3) | 0.51 |
| Δ 12-24 weeks | -1.0 ± 1.0 | -0.3 ± 1.8 | 0.7 (-0.7, 2.1) | 0.29 |
| **Stair climb time (s)** |  |  |  |  |
| Baseline | 3.7 ± 0.6 | 4.3 ± 1.8 |  |  |
| ﻿Δ 0-12 weeks | 0.02 ± 0.5 | -0.3 ± 0.9 | -0.3 (-0.9, 0.3) | 0.30 |
| Δ 12-24 weeks | -0.1 ± 0.4 | 0.1 ± 0.7 | 0.2 (-0.3, 0.8) | 0.38 |
| **Gait speed (m/s)** |  |  |  |  |
| Baseline | 0.89 ± 0.2 | 0.84 ± 0.2 |  |  |
| ﻿Δ 0-12 weeks | 0.01 ± 0.1 | 0.01 ± 0.2 N=14 | 0.01 (-0.1, 0.1) | 0.91 |
| Δ 12-24 weeks | 0.1 ± 0.1 N=8 | 0.02 ± 0.2 N=12 | -0.1 (-0.2, 0.1) | 0.24 |
| **400 metre walk time (s)** |  |  |  |  |
| Baseline | 241.9 ± 28.1 | 257.7 ± 39.5 |  |  |
| ﻿Δ 0-12 weeks | -11.1 ± 14.2 | -1.3 ± 15.5 | 9.8 (-2.8, 22.5) | 0.12 |
| Δ 12-24 weeks | -1.0 ± 10.8 | -2.9 ± 23.6 N=12 | -1.9 (-19.7, 15.9) | 0.82 |
| **SPPB score** |  |  |  |  |
| Baseline | 11.0 ± 2.1 | 11.3 ± 1.0 |  |  |
| ﻿Δ 0-12 weeks | 0.4 ± 1.1 N=10 | 0.3 ± 0.8 | -0.1 (-0.8, 0.7) | 0.86 |
| Δ 12-24 weeks | 0.0 ± 0.0 N=8 | 0.1 ± 0.6 | 0.1 (-0.4, 0.6) | 0.74 |

Baseline values are mean ± SD. Net differences are means with 95% confidence intervals. Bold indicates statistical significance (P<0.05). HOMA-IR - homeostatic model assessment of insulin resistance; HDL - high-density lipoprotein; LDL - low-density lipoprotein; BMI – body mass index; ALM – appendicular lean mass; ALM/H^2^ – appendicular lean mass/ height^2^; SPPB – short physical performance battery. Placebo: ﻿Δ 0-12 weeks N=11, Δ 12-24 weeks N=9; Vitamin D: Δ 12-24 weeks N=13.

**Supplementary Table S6**. Mean baseline values and within- and between-group changes in blood biochemistry, body composition and physical function after 12 and 24 weeks in participants with obesity randomized to placebo or vitamin D.

|  | Placebo  N= 12 | Vitamin D  N=11 | Net Difference  (95%CI) | Group x Time  (P-value) |
| --- | --- | --- | --- | --- |
| **Blood biochemistry** |  |  |  |  |
| **25-hydroxyvitamin D (nmol/L)** |  |  |  |  |
| Baseline | 40.3 ± 11.4 | 43 ± 12.4 |  |  |
| ﻿Δ 0-12 weeks | -0.9 ± 10.1 | 38.7 ± 13.1 | **39.6 (28.2, 51.0)** | **<0.001** |
| Δ 12-24 weeks | 8.3 ± 11.9 | 4.8 ± 8.1 | -3.5 (-13.8, 6.8) | 0.48 |
| **Fasting glucose (mmol/L)** |  |  |  |  |
| Baseline | 5.7 ± 1 | 6.0 ± 1.4 |  |  |
| ﻿Δ 0-12 weeks | -0.04 ± 0.6 | 0.6 ± 1.9 | 0.7 (-0.7, 2.0) | 0.32 |
| Δ 12-24 weeks | 0.1 ± 0.6 N=6 | 0.7 ± 1.0 | 0.5 (-0.4, 1.5) | 0.25 |
| **Fasting insulin (mUL)** |  |  |  |  |
| Baseline | 12.8 ± 8.3 | 12.6 ± 4.8 |  |  |
| ﻿Δ 0-12 weeks | 2 ± 4.4 | 1.2 ± 5.4 | -0.8 (-5.6, 4.0) | 0.73 |
| Δ 12-24 weeks | -2.8 ± 3.8 | -0.2 ± 4.6 | 2.6 (-1.9, 7.1) | 0.24 |
| **HOMA-IR (units)** |  |  |  |  |
| Baseline | 3.4 ± 2.6 | 3.4 ± 1.8 |  |  |
| ﻿Δ 0-12 weeks | 0.6 ± 1.4 | 1.3 ± 3.5 | 0.6 (-2.0, 3.2) | 0.63 |
| Δ 12-24 weeks | -0.9 ± 1.2 N=6 | 0.8 ± 2.6 | 1.7 (-0.8, 4.2) | 0.17 |
| **Triglycerides (mmol/L)** |  |  |  |  |
| Baseline | 1.8 ± 1.1 | 1.6 ± 0.6 |  |  |
| ﻿Δ 0-12 weeks | 0.1 ± 0.6 | 0.2 ± 0.6 | 0.1 (-0.5, 0.7) | 0.69 |
| Δ 12-24 weeks | -0.2 ± 0.5 | -0.03 ± 0.2 | 0.2 (-0.3, 0.6) | 0.44 |
| **HDL (mmol/L)** |  |  |  |  |
| Baseline | 1.4 ± 0.2 | 1.4 ± 0.3 |  |  |
| ﻿Δ 0-12 weeks | -0.03 ± 0.2 | -0.04 ± 0.1 | -0.01 (-0.2, 0.2) | 0.93 |
| Δ 12-24 weeks | 0.1 ± 0.4 | 0.02 ± 0.2 | -0.1 (-0.3, 0.2) | 0.70 |
| **LDL (mmol/L)** |  |  |  |  |
| Baseline | 3.3 ± 0.6 | 3.3 ± 1.6 |  |  |
| ﻿Δ 0-12 weeks | -0.2 ± 0.4 | -0.3 ± 0.4 | -0.1 (-0.5, 0.3) | 0.52 |
| Δ 12-24 weeks | 0.1 ± 0.3 | 0.2 ± 0.5 | 0.1 (-0.3, 0.6) | 0.52 |
| **Body composition** |  |  |  |  |
| **Weight (kg)** |  |  |  |  |
| Baseline | 98.8 ± 14.2 | 97.6 ± 12.2 |  |  |
| ﻿Δ 0-12 weeks | -0.02 ± 4.9 | -0.9 ± 1.6 | -0.9 (-4.3, 2.6) | 0.61 |
| Δ 12-24 weeks | -0.2 ± 1.2 | 0.2 ± 2.5 | 0.4 (-1.7, 2.6) | 0.67 |
| **BMI (kg/m^2^)** |  |  |  |  |
| Baseline | 36.7 ± 5.3 | 34.3 ± 3.4 |  |  |
| ﻿Δ 0-12 weeks | 0.04 ± 1.8 | -0.3 ± 0.8 | -0.4 (-1.7, 1.0) | 0.57 |
| Δ 12-24 weeks | -0.03 ± 0.6 | 0.2 ± 1.0 | 0.2 (-0.6, 1.1) | 0.57 |
| **Waist circumference (cm)** |  |  |  |  |
| Baseline | 112.2 ± 12 | 113.8 ± 8.1 |  |  |
| ﻿Δ 0-12 weeks | -1.1 ± 5.9 | -2.2 ± 1.5 | -1.1 (-5.2, 2.9) | 0.57 |
| Δ 12-24 weeks | 3.3 ± 5.9 | -5.2 ± 6.4 | **-8.4 (-14.9, -2.0)** | **0.01** |
| **Hip circumference (cm)** |  |  |  |  |
| Baseline | 121.9 ± 8.7 | 118.8 ± 12.5 |  |  |
| ﻿Δ 0-12 weeks | 0.3 ± 3.6 | -1.1 ± 3.0 | -1.4 (-4.6, 1.8) | 0.36 |
| Δ 12-24 weeks | -0.4 ± 2.7 | 1.6 ± 2.6 | 2.0 (-0.8, 4.8) | 0.15 |
| **Waist Hip Ratio** |  |  |  |  |
| Baseline | 0.9 ± 0.1 | 1.0 ± 0.1 |  |  |
| ﻿Δ 0-12 weeks | -0.01 ± 0.1 | -0.01 ± 0.02 | -0.003 (-0.04, 0.04) | 0.88 |
| Δ 12-24 weeks | 0.03 ± 0.1 | -0.1 ± 0.05 | **-0.1 (-0.1, -0.03)** | **<0.001** |
| **ALM (kg)** |  |  |  |  |
| Baseline | 24 ± 6.4 | 24.9 ± 6.1 |  |  |
| ﻿Δ 0-12 weeks | 0.2 ± 2.0 | 0.03 ± 0.8 | -0.2 (-1.6, 1.2) | 0.77 |
| Δ 12-24 weeks | 0.2 ± 0.5 | 0.02 ± 0.4 | -0.2 (-0.6, 0.2) | 0.32 |
| **ALM/height^2^ (kg)** |  |  |  |  |
| Baseline | 8.8 ± 1.3 | 8.6 ± 1.3 |  |  |
| ﻿Δ 0-12 weeks | 0.1 ± 0.7 | 0.02 ± 0.3 | -0.1 (-0.6, 0.4) | 0.75 |
| Δ 12-24 weeks | 0.1 ± 0.2 | 0.005 ± 0.1 | -0.1 (-0.2, 0.1) | 0.37 |
| **Fat mass (kg)** |  |  |  |  |
| Baseline | 42.6 ± 9.6 | 40.8 ± 10.1 |  |  |
| ﻿Δ 0-12 weeks | 0.2 ± 2.7 | -0.7 ± 2.1 | -0.9 (-3.4, 1.6) | 0.45 |
| Δ 12-24 weeks | -0.3 ± 1.1 | 0.4 ± 2.6 | 0.7 (-1.7, 3.1) | 0.54 |
| **Body fat percentage** |  |  |  |  |
| Baseline | 45.3 ± 7.8 | 43.7 ± 9.2 |  |  |
| ﻿Δ 0-12 weeks | -0.1 ± 1.3 | -0.4 ± 1.7 | -0.4 (-1.9, 1.2) | 0.64 |
| Δ 12-24 weeks | 0.1 ± 1.0 | 0.4 ± 1.7 | 0.3 (-1.4, 1.9) | 0.76 |
| **Visceral adipose tissue (cm^2^)** |  |  |  |  |
| Baseline | 184.7 ± 53.7 | 168.8 ± 42.1 |  |  |
| ﻿Δ 0-12 weeks | -3.4 ± 23.2 | -11.7 ± 20.4 | -8.3 (-30.8, 14.3) | 0.45 |
| Δ 12-24 weeks | 0.7 ± 18.5 | -3.4 ± 22.0 | -4.1 (-27.2, 18.9) | 0.71 |
| **Calf muscle density (mg/cm^3^)** |  |  |  |  |
| Baseline | 73.3 ± 3.0 | 72.8 ± 2.5 |  |  |
| ﻿Δ 0-12 weeks | -0.6 ± 0.9 N=8 | -0.4 ± 1.1 N=9 | 0.1 (-1.0, 1.3) | 0.79 |
| Δ 12-24 weeks | -0.1 ± 1.9 N=5 | 0.1 ± 1.0 N=8 | 0.2 (-1.7, 2.1) | 0.82 |
| **Physical function** |  |  |  |  |
| **Hand grip strength (kg)** |  |  |  |  |
| Baseline | 30.4 ± 16 | 36.9 ± 13.9 |  |  |
| ﻿Δ 0-12 weeks | 0.8 ± 4.2 N=7 | 0.6 ± 3.2 | -0.2 (-4.0, 3.6) | 0.92 |
| Δ 12-24 weeks | -1.7 ± 4.3 N=6 | -0.7 ± 1.9 | 1.0 (-2.3, 4.4) | 0.51 |
| **Upper-limb relative strength** |  |  |  |  |
| Baseline | 9.7 ± 2.5 | 11.2 ± 1.8 |  |  |
| ﻿Δ 0-12 weeks | 0.3 ± 1.8 N=7 | 0.2 ± 1.0 | -0.2 (-1.6, 1.3) | 0.79 |
| Δ 12-24 weeks | -1.0 ± 1.8 N=6 | -0.2 ± 0.7 | 0.8 (-0.5, 2.1) | 0.23 |
| **Chair stand time (s)** |  |  |  |  |
| Baseline | 10.9 ± 2.3 | 9.8 ± 2.1 |  |  |
| ﻿Δ 0-12 weeks | -1.5 ± 2.7 | -1.3 ± 1.5 | 0.2 (-1.8, 2.3) | 0.82 |
| Δ 12-24 weeks | -1.7 ± 2.2 | 0.1 ± 1.6 N=9 | 1.8 (-0.3, 3.8) | 0.08 |
| **Stair climb time (s)** |  |  |  |  |
| Baseline | 4.9 ± 1.5 | 4.0 ± 0.6 |  |  |
| ﻿Δ 0-12 weeks | 0.4 ± 0.9 | -0.2 ± 0.7 | -0.5 (-1.3, 0.3) | 0.19 |
| Δ 12-24 weeks | 0.1 ± 1.2 | 0.4 ± 1.0 | 0.3 (-0.9, 1.4) | 0.60 |
| **Gait speed (m/s)** |  |  |  |  |
| Baseline | 0.82 ± 0.2 | 0.96 ± 0.2 |  |  |
| ﻿Δ 0-12 weeks | -0.03 ± 0.1 | -0.04 ± 0.2 | -0.01 (-0.2, 0.1) | 0.82 |
| Δ 12-24 weeks | 0.03 ± 0.2 | 0.01 ± 0.2 N=8 | -0.02 (-0.2, 0.2) | 0.84 |
| **400 metre walk time (s)** |  |  |  |  |
| Baseline | 301.6 ± 61.3 | 267 ± 27.8 |  |  |
| ﻿Δ 0-12 weeks | -4.4 ± 23.1 | -4.0 ± 15.5 | 0.4 (-18.4, 19.3) | 0.96 |
| Δ 12-24 weeks | 7.0 ± 13.4 N=6 | 8.2 ± 26.5 | 1.2 (-24.0, 26.4) | 0.92 |
| **SPPB score** |  |  |  |  |
| Baseline | 10.8 ± 1.1 | 11.6 ± 0.9 |  |  |
| ﻿Δ 0-12 weeks | 0.3 ± 0.9 | -0.4 ± 1.3 | -0.7 (-1.8, 0.3) | 0.16 |
| Δ 12-24 weeks | 0.9 ± 1.2 | 0.1 ± 1.9 | -0.8 (-2.5, 1.0) | 0.37 |

Baseline values are mean ± SD. Net differences are means with 95% confidence intervals. Bold indicates statistical significance (P<0.05). HOMA-IR - homeostatic model assessment of insulin resistance; HDL - high-density lipoprotein; LDL - low-density lipoprotein; BMI – body mass index; ALM – appendicular lean mass; ALM/H^2^ – appendicular lean mass/ height^2^; SPPB – short physical performance battery. Placebo: ﻿Δ 0-12 weeks N=9, Δ 12-24 weeks N=7; Vitamin D: ﻿Δ 0-12 weeks N=10, Δ 12-24 weeks N=10.
